# Double-strand break toxicity is chromatin context independent

**DOI:** 10.1093/nar/gkac758

**Published:** 2022-09-15

**Authors:** Anoek Friskes, Lisa Koob, Lenno Krenning, Tesa M Severson, Emma S Koeleman, Xabier Vergara, Michael Schubert, Jeroen van den Berg, Bastiaan Evers, Anna G Manjón, Stacey Joosten, Yongsoo Kim, Wilbert Zwart, René H Medema

**Affiliations:** Oncode Institute, Division of Cell Biology, the Netherlands Cancer Institute, Plesmanlaan 121, 1066 CX Amsterdam, The Netherlands; Oncode Institute, Division of Cell Biology, the Netherlands Cancer Institute, Plesmanlaan 121, 1066 CX Amsterdam, The Netherlands; Oncode Institute, Division of Cell Biology, the Netherlands Cancer Institute, Plesmanlaan 121, 1066 CX Amsterdam, The Netherlands; Oncode Institute, Division of Oncogenomics, the Netherlands Cancer Institute, Plesmanlaan 121, 1066 CX Amsterdam, The Netherlands; Oncode Institute, Division of Cell Biology, the Netherlands Cancer Institute, Plesmanlaan 121, 1066 CX Amsterdam, The Netherlands; Oncode Institute, Division of Cell Biology, the Netherlands Cancer Institute, Plesmanlaan 121, 1066 CX Amsterdam, The Netherlands; Oncode Institute, Division of Gene Regulation, the Netherlands Cancer Institute, Plesmanlaan 121, 1066 CX Amsterdam, The Netherlands; Oncode Institute, Division of Cell Biology, the Netherlands Cancer Institute, Plesmanlaan 121, 1066 CX Amsterdam, The Netherlands; Oncode Institute, Division of Cell Biology, the Netherlands Cancer Institute, Plesmanlaan 121, 1066 CX Amsterdam, The Netherlands; Oncode Institute, Division of Molecular Carcinogenesis and NKI Robotics and Screening Center, The Netherlands Cancer Institute, Plesmanlaan 121, 1066 CX Amsterdam, The Netherlands; Oncode Institute, Division of Cell Biology, the Netherlands Cancer Institute, Plesmanlaan 121, 1066 CX Amsterdam, The Netherlands; Oncode Institute, Division of Oncogenomics, the Netherlands Cancer Institute, Plesmanlaan 121, 1066 CX Amsterdam, The Netherlands; Oncode Institute, Division of Oncogenomics, the Netherlands Cancer Institute, Plesmanlaan 121, 1066 CX Amsterdam, The Netherlands; Oncode Institute, Division of Oncogenomics, the Netherlands Cancer Institute, Plesmanlaan 121, 1066 CX Amsterdam, The Netherlands; Oncode Institute, Division of Cell Biology, the Netherlands Cancer Institute, Plesmanlaan 121, 1066 CX Amsterdam, The Netherlands

## Abstract

Cells respond to double-strand breaks (DSBs) by activating DNA damage response pathways, including cell cycle arrest. We have previously shown that a single double-strand break generated via CRISPR/Cas9 is sufficient to delay cell cycle progression and compromise cell viability. However, we also found that the cellular response to DSBs can vary, independent of the number of lesions. This implies that not all DSBs are equally toxic, and raises the question if the location of a single double-strand break could influence its toxicity. To systematically investigate if DSB-location is a determinant of toxicity we performed a CRISPR/Cas9 screen targeting 6237 single sites in the human genome. Next, we developed a data-driven framework to design CRISPR/Cas9 sgRNA (crRNA) pools targeting specific chromatin features. The chromatin context was defined using ChromHMM states, Lamin-B1 DAM-iD, DNAseI hypersensitivity, and RNA-sequencing data. We computationally designed 6 distinct crRNA pools, each containing 10 crRNAs targeting the same chromatin state. We show that the toxicity of a DSB is highly similar across the different ChromHMM states. Rather, we find that the major determinants of toxicity of a sgRNA are cutting efficiency and off-target effects. Thus, chromatin features have little to no effect on the toxicity of a single CRISPR/Cas9-induced DSB.

## INTRODUCTION

DNA damage can be caused by irradiation, chemicals, or during essential cellular processes such as transcription or DNA replication. Cells can recognize and repair the damage through a complex interplay of many proteins that not only sense and repair the damage, but can also trigger a cell cycle arrest (1). When encountering a DSB, the cell continues the cell cycle once the break is repaired, or it permanently exits the cell cycle (2). A cell has three major ways to permanently exit the cell cycle: it can enter a senescent state, or the cell can undergo cell death, via apoptosis or necrosis (3). The decision on whether a cell continues to proliferate is mainly thought to be regulated via differential activation of cell cycle checkpoints (4–6). Cell cycle checkpoints are activated when the cell encounters broken DNA-ends and facilitate the arrest of cells at the G1/S or G2/M transition to provide time for DNA repair to occur (7). When the checkpoint signal is high and/or maintained over a longer time, the outcome is more likely to be irreversible (8).

After activation of cell cycle checkpoints, the DNA damage response (DDR) promotes the recruitment of most of the repair factors. Damage can be repaired through several canonical repair pathways. Classical non-homologous end joining (c-NHEJ) directly ligates the break ends and is active throughout the cell cycle (9). Alternatively, broken ends can be resected and repaired via single-strand annealing (SSA), microhomology-mediated repair (MMEJ), or homologous recombination (HR), which is mainly active in S/G2 (10–12). Repair pathway choice and efficiency is in part dependent on the cell cycle stage, the local chromatin context, sequence and 3D genome organization (13–16). Topologically associating domains (TADs) are important for 3D genome organization and are functional units of the DNA damage response and crucial for establishing an environment that enables efficient repair of damage (17,18). This mainly involves chromatin restructuring aimed to facilitate the accessibility of repair factors, but this can differ per genomic location (19). These findings have sparked a growing interest in the role of chromatin in determining cell fate after a DSB.

Studying how chromatin context influences cell fate after encountering DSBs is difficult when using DNA damaging agents due to the random nature of break induction. To overcome the obstruction of random DNA damage induction, the implementation of clustered regularity interspaced palindromic repeat (CRISPR)/Cas9 in human cells allows for location-specific studies (20). Consequently, CRISPR/Cas9 has been used to study location-dependent effects on repair pathway usage (16) and cell fate decisions (21–23). The toxicity of DSBs might differ between genomic loci (21), thus individual DSBs could potentially drive different cell fate outcomes depending on their genomic location. For example, repair of rDNA repeats via recombination can result in loss or gain of rDNA repeats, demonstrating that recruitment of HR factors to DSBs in rDNA repeats presents a liability to the genomic integrity of a cell (21,24).

Although most studies are focused on the influence of local chromatin, DNA sequence, and stage of the cell cycle on repair pathway usage, the effects of local chromatin context on cell cycle delay after a single DSB have not been assessed thus far. Here we present two parallel approaches to investigate the influence of chromatin context on the cell cycle delay (toxicity) after a single DSB that is introduced by CRISPR/Cas9. Our assays monitor the capacity of single cutting sgRNAs to attenuate cell division. This could occur either via a cell cycle delay (23), cell death, a cell cycle exit, or the induction of senescence. We refer to this attenuation of cell proliferation as the ‘toxicity’ of the sgRNA, which is not to be taken as evidence that this sgRNA results in cell death. First, we performed a CRISPR/Cas9-based screen with 6237 sgRNAs that were designed to induce only a single DSB and assessed the outgrowth of the cells to identify the more toxic sgRNAs. Second, we composed small pools of sgRNAs that generate a single DSB in distinct genomic locations with specific chromatin features and assessed DSB-induction and cellular outgrowth. Both approaches show that the toxicity of a CRISPR/Cas9 induced DSB is independent of chromatin features at the break site. Instead, we show that the differential toxicity of sgRNAs is for the largest part driven by off-target induction of DSBs and the editing efficiency of the sgRNA.

## MATERIALS AND METHODS

### Cell lines

Retinal pigment epithelial (RPE-1) hTERT cell lines were grown in DMEM:F12 GlutaMAX medium (Gibco) containing 1% penicillin–streptomycin and 6% FBS. Cells were cultured at 37°C in a 5% CO_2_ atmosphere with 21% oxygen. Chemicals used in this study: SHIELD-1 (Aobious, 1 uM) and Doxycycline (Sigma, 1 mM), Puromycin (20 ug/ml), Blasticidine (10 ug/ml). Cell lines were determined to be free from mycoplasma contamination regularly using DAPI staining.

### Transfections

#### DNA transfection and lentiviral production

The pLentiGuide-puromycin (#117986; Addgene) was used and pCW-Cas9 was a gift from Eric Lander and David Sabatini (#50661; Addgene). Lentiviral plasmids were co-transfected into HEK293T cells with the lentiviral packaging plasmids pMD2.G and psPAX2 (#12260 and #12259; Addgene). 5 × 10^6^ HEK293T cells were seeded into a 10 cm culture dish the day before transfection. For each 10 cm dish, the DNA was diluted in 500 ul OptiMEM (Thermo Fisher Scientific): 4 μg lentiviral vector, 4 μg psPAX2, and 4 μg pMD2.G. Separately, 36 μl of FuGene was diluted into 500 ul OptiMEM and incubated at room temperature for 5 min. After that, the FuGene and DNA mixtures were combined, and incubated at room temperature for 20 min. The transfection mixture was then added dropwise to the 10 cm dish. Viral particles were harvested 48h after the media change and filtered through a 0.45 μm PVDF syringe filter. The filtered supernatant was used directly to infect cells and aliquots were frozen and stored at −80°C. For infection, lentiviral particles were diluted in complete growth media supplemented with 8 μg/ml polybrene (Sigma-Aldrich) and added to cells.

#### TracrRNA:crRNA

Alt-R crRNAs were generated by Integrated DNA technologies (IDT) for all 60 crRNA sequences of the pools (for sequences, see Supplementary Table S4). TracrRNA:crRNA duplex was transfected according to manufacturer's protocol (https://www.idtdna.com/pages/products/crispr-genome-editing/alt-r-crisprcas9-system). Briefly, tracrRNA and crRNA were hybridized at 95ºC for 5 min in a 1:1 ratio. In the meantime, RNAiMAX was diluted in OptiMEM. After hybridization, the mixture was combined, and incubated at room temperature for 20 min. The transfection mixture was then added dropwise to the dish.

### Cloning of lentiGuide-Puro-T2A-sfGFP/mCherry

To create pLentiGuide-puro (Addgene vector 52963) co-expressing sfGFP of mCherry, pLentiGuide-puro was first linearized using MluI. T2A-mCherry and T2A-sfGFP were PCR amplified using the following forward primer 5′-acctggtgcatgacccgcaagcccggtgccGGAGGATCGGGAGAGGGCAGAGGAAGTCT-3′ and the reverse primer 5′-tttgtaatccagaggttgattgtcgacttaacgcgtttaTTTGTAGAGCTCATCCATGCC-3′ for sfGFP and 5′tttgtaatccagaggttgattgtcgacttaacgcgtttacttgtacagctcgtccatgcc-3′ for mCherry. These PCR products were assembled into the recipient vector using Gibson assembly, and the resulting plasmids were verified by sanger sequencing. Introduction of the sgRNAs into these plasmids was performed as described for the original pLentiGuide-Puro vector. The resulting vectors (plentiGuide-Puro-T2AsfGFP), plentiGuide-Puro-T2A-mCherry) were sequence verified and plentiGuide-Puro-T2A-sfGFP was used for construction of the SSC library.

### Single cutter library design and cloning

We generated 1.0 × 10^8^ random 20 nucleotide sequences and appended an NGG on the 5’ end of the random sequences to create sgRNAs targeting sequences. We used the iKRUNC package (http://github.com/Toverkwark/ikrunc) previously described by Evers et al. to select sgRNA with a single unique target in the human genome (25). Briefly, we aligned these sequences to the human genome (Hg19) and kept the sgRNA which perfectly aligned. Next, we discarded the sgRNA which aligned to the genomic location with up to and including three mismatches. One hundred non-targeting sgRNA controls were added from Wang et al. (26). An additional Guanine was added to the 5’ of the sgRNA to ensure expression from the U6 promoter. The complete library can be found in Supplementary Table S1.

To clone the single cutter library, pools of oligonucleotides were ordered from CustomArray (Bothell, WA) containing sgRNA sequences flanked by 20–30 nt of overlapping vector sequence. Cloning was performed similar to Sanjana et al. (27). Briefly, the custom array was amplified by PCR with ArrayF 5′-TAACTTGAAAGTATTTCGATTTCTTGGCTTTATATATCTTGTGGAAAGGACGAAACACCG-3′ & ArrayR 5′-ACTTTTTCAAGTTGATAACGGACTAGCCTTATTTTAACTTGCTATTTCTAGCTCTAAAAC-3′ with Phusion polymerase (NEB) with 63ºC annealing temperature. The PCR product was purified and ligated with Gibson Assembly into a BsmBI digested plentiGuide-Puro-T2AsfGFP. The library was transformed into *Escherichia coli* 10G electrocompetent cells (Lucigen) and plated on 15cm LB agar with carbenicillin selection (50 ug/ml). Plates were scraped, pelleted and plasmid DNA was purified with Qiagen Maxiprep kit.

CustomArray order: GGAAAGGACGAAACACCGNNNNNNNNNNNNNNNNNNNNGTTTTAGAGCTAGAAATAGCAAGTTAAAATAAGGC.

### Screen

To screen the toxicity of DSBs at a single locus, RPE-1 wild-type, RPE-1 Cas9 and RPE-1 Cas9 p53^–/–^ cells were infected with a custom designed sgRNA library (see Methods above). Cells were infected over-night in the presence of hexadimethrine bromide (polybrene, 8μg/ml), at MOI <1. The percentage of infected cells was determined by FACS at 4 days after infection, using cells that were grown in the absence of a selection agent (Supplementary Figure S1D). To select for infected cells, puromycin (10 ug/ml) was added 2 days post infection. After 2 days of selection, when infected cells accounted for >90% of the population, a reference sample (*t*_0_) was collected with a minimum of 1000× library coverage (8–25 × 10^6^ cells). Per condition 6.5 × 10^6^ cells were re-plated into medium containing puromycin. Cells were collected on 8- and 16-day post-infection, again at a minimum of 1000x library coverage. Genomic DNA was isolated from frozen cell pellets, the sgRNA containing region was amplified, barcodes were added and the sample was processed for sequencing on Illumina MiSeq (for primer sequences, see Supplementary Table S4). Sequencing reads were aligned to the custom library and counted. Read counts of sgRNAs were normalized against total read counts per sample. For all conditions (wild-type, Cas9-expressing wild-type, Cas9-expressing p53^–/–^ RPE-1 cells), we calculated the differential abundance of all replicates shortly after the infection (4-day time point) and after selection was finished (8 day or 16-day time point). For this, DESeq2 1.31.3 (R 4.0.5) was used to estimate the library size, by utilizing a paired design with default parameters and control guides only (CTRL prefix). Fold-change data comparing day 4/8 and day 4/16 can be found in Supplementary Tables S2 and S3.

### Design of CRISPR-Cas9 crRNA pools

The 6 pools of sgRNAs were designed by integrating ChIP-sequencing data, Lamin-B1 DAM-iD, DNAseI hypersensitivity and RNA-sequencing data from RPE-1 cells using an analysis pipeline in R, available from Github (https://github.com/eskoeleman/gRNA_environment). In short, it comprises a sgRNAinfo script which requires a library of sgRNA locations as an input and returns an output table containing location specific data for each dataset. Next, these tables are used in the sgRNAfilter script, in which filtering thresholds are defined to select sgRNAs in a specific environment. The pipeline combines publicly available datasets for the analysis. ChromHMM analysis of the ChIP-sequencing data of H3K27me3, H3K36me3, H3K4me3 and H3K4me1 was performed previously (28). For H3K9me3 an additional ChIP-analysis dataset from GEO was used (GSM3105086). Additional ChIP-sequencing on H3K9me3, H3K27Ac and H2A.Z was performed in this study and are deposited in GEO (GSE210402). The Lamin-B1 DAM-iD data was analysed using a hidden Markov model to call LADs and iLADs and was retrieved from the 4DNucleome consortium (accession number: 4DNESHGTQ73M) (29–31). DNaseI hypersensitive sites were identified using DNaseI data from ENCODE (dataset: ENCSR000EON, file: ENCFF128BPC) (32). RefSeq data was acquired from NCBI (33). RNA-sequencing data of RPE-1 wild-type cells was previously performed in the lab and the dataset from GEO was used (GSE163315) to asses gene expression and to define intronic locations. For selection of the six pools, the CRISPR/Cas9 sgRNA library from the screen was used, which contained 6237 single-cutting sgRNAs.

**Figure 1.**
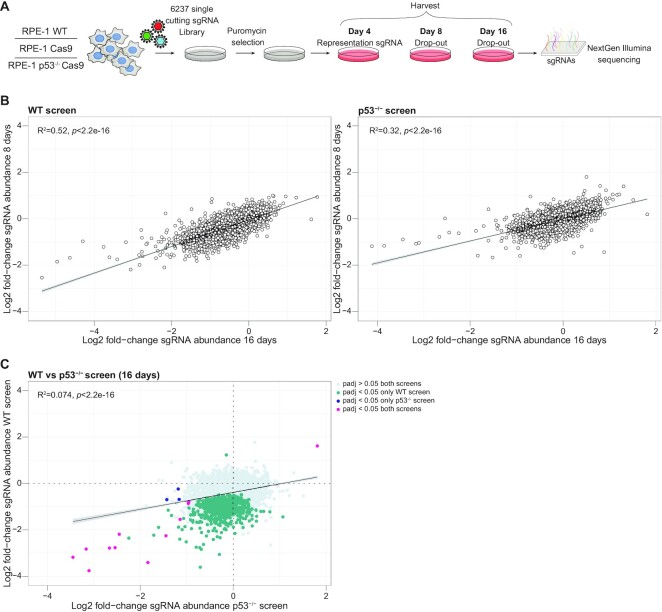
CRISPR/Cas9 screens to identify toxic DSB locations in the genome. (**A**) Schematic representation of the genome-wide CRISPR/Cas9 screen to identify the toxicity of DSBs, using a library of 6237 single cutting sgRNAs in RPE-1 WT, RPE-1 cells expressing Cas9 and p53^-/-^ RPE-1 cells expressing Cas9. (**B**) Correlation plot showing the fold-change of sgRNA abundance after 8 and 16 days, normalized to control in p53-wildtype and p53-deficient cells. Depicted is the mean of three independent screen replicates. (**C**) Correlation plot showing the fold-change of sgRNA abundance at 16 days between p53-wildtype and p53- deficient cells. Indicating the sgRNAs dropping out in both screens (*P*adj < 0.05, magenta), only in the p53-wildype screen (*P*adj < 0.05, green) or only dropping out in the p53- deficient screen (*P*adj < 0.05, blue).

All sgRNAs (SCC_screen) classified in ChromHMM states (Hg19 and Hg38) can be found in Supplementary Table S5. In the sgRNAinfo script, integrated analysis of all datasets was performed using a window of 4 kb around the sgRNA binding site. Following, the sgRNAfilter script was used to define filtering parameters for each pool, as shown in Figure 1C. For pool 2, localized at promoter/enhancer sites, at least 65% of the 4kb window was required to be called as ChromHMM state 2, provided that the breaksite itself was also called as state 2. Additionally, DNaseI peak height was required to exceed 0.2, over 90% of the 4 kb window was called as iLAD and each location was defined as genic and intronic based on RefSeq (33). Intronic locations were chosen for all genic locations, to minimize toxic genic cell responses. Pool 4 marked enhancer sites were selected on ChromHMM state 4 and did not include filtering of DNaseI. Locations were >99% in iLADs. Pool 4 contained two sgRNAs in genic regions and eight sgRNAs in non-genic regions. Next, pool 7 marked gene bodies and was >99% located in ChromHMM state 7 within the 4 kb window. The highest DNaseI peak in the region had to exceed >6%, DAMID >90% iLAD and all locations were both genic and intronic. Pool 8a and 8b were selected similarly, except for DAMID, which was >99% in iLAD for pool 8a and >99% in LAD for pool 8b. Furthermore, both were located >99% in state 8 and in non-genic regions. Finally, pool 9 was associated with condensed chromatin and was defined by having an average peak height of >5.5 based on the H3K9me3 ChIP-sequencing data. Additionally, each location was >99% in LAD, nongenic and was confirmed to be state 9 in the ChromHMM data used for defining the other pools. From the list of sgRNAs that was left after filtering, 10 sgRNAs were selected for each pool, considering that a maximum of two locations per chromosome was allowed within a pool.

**Table:** 

Step		Pool2	Pool4	Pool7	Pool 8a	Pool8b	Pool9
1.	ChromHMM (% of window)	>65% state 2	> 50% State 4	>99% State 7	>99% State 8	>99% State 8	Weighted peak height (>5.5)
2.	DNaseI (max. peak height within window)	>0.2		<0.06			
3.	DAM-iD (% of window)	>90% iLAD	>99% iLAD	>90% iLAD	>99% iLAD	>99% LAD	>99% LAD
4.	RefSeq (annotation)	Genic Intronic	Mixed	Genic Intronic	Non-genic	Non-genic	Non-genic

### Outgrowth assays

#### Colony forming assay

RPE-1 iCut cells (previously described (23)) were transfected with crRNAs at a final concentration of 20 nM. 24h later, cells were trypsinized, counted and 250 single cells were plated in six-well plates in three technical replicates. Cells were allowed to grow out for 7 days. Plates were fixed in 80% Methanol and stained with 0.2% Crystal Violet. Plates were scanned and analyzed with ImageJ software (NIH) and relative cell survival plots were generated.

#### Outgrowth assay

For the outgrowth experiments, 2000 RPE-1 iCut cells were plated in each well of a 96-well plate. The next day, these cells were transfected in two technical replicates with crRNAs at a final concentration of 20 nM. 24h later cells were trypsinized, counted and all the cells were replated in a 24-well plate. Cells were allowed to grow out for 6 days, followed by fixation in 80% Methanol and stained with 0.2% Crystal Violet. To measure outgrowth, 50 ul of 10% acidic acid (in water) was added followed by addition of 150 ul of water. Absorption was measures using an Epoch Spectrophotometer from BioTek using the Gen5 3.05 software.

### Competition growth assay

For competition growth assays, RPE-1 wild-type cells were either infected with a viral construct containing a control sgRNA co-expressing mCherry or containing the targeted sgRNA co-expressing sfGFP fluorophore (see methods, section Cloning of lentiGuide-Puro-T2A-sfGFP/mCherry). The two populations were then mixed at a 1:1 ratio and seeded into duplicate wells. After seeding, one of the wells was Cas9-infected and after 1 day washed with PBS followed by Blasticidine selection. Three days later, each well was washed, trypsinized and 50% of the cells were analyzed on a flow cytometer to determine the mCherry:sfGFP ratio. This was repeated 12 days later. The value obtained from the Cas9 infected well was then normalized to the untreated well to determine the fold change in mCherry:sfGFP positive cells.

### Foci imaging (staining + imaging)

For immunofluorescence (IF) staining, 2000 cells were plated in 96-well plates and the next day transfected with the crRNAs. 24 hours later, cells were fixed and permeabilized with 3.7% formaldehyde + 0.5% Triton-X100 for 20 min, followed by 3 × 5 min wash steps with PBS + 0.1% Tween 20 (PBS-T). Cells were incubated at room temperature (RT) for 1.5 h with primary antibody in PBS-T, washed three times with PBS-T and incubated with DAPI complemented with secondary antibody in PBS-T for 2 h at RT. The following antibody was used in this study: anti-53BP1 (1:1000). Secondary antibody used for IF analysis was: anti-mouse Alexa 549 (1:1000). DAPI was used in a final concentration 1 μg/ml. Images were obtained using a Lionheart FX automated Live cell Imager from BioTek using a 20× airlens 0.45 NA or a THUNDER Imager 3D Cell Culture van Leica 63× oil lens: Obj. HC PL APO 63×/1.40–0.60 OIL 11506349. DNA damage foci were evaluated in ImageJ, using an in-house developed macro that enabled automatic and objective analysis (previously described (23)).

### ChIP-sequencing of RPE-1 hTERT cells

Chromatin immunoprecipitations (ChIP) were performed as described previously with minor adjustments (34). For ChIP of histone marks, approximately 7 million cells, 50μL of Protein A magnetic beads (Invitrogen) and 5μg of antibody were used. Antibodies were H3K27ac (Actif Motif #39133), H3K9me3 (ab8898), H2AZ (ab4174). ChIP-seq samples were processed for library preparation (Part# 0801-03003, KAPA Biosystems kit) and sequenced with Illumina NovaSeq6000 (54bp paired-end reads). Mapped reads were filtered based on mapping quality of 20 using samtools version 0.1.19. Genome browser snapshots, heatmaps and density plots were generated using EaSeq (http://easeq.net).

### ChIP-seq data analysis

For Hg19 ChromHMM generation, H3K27me3 (Millipore 07-449, GSM6429706), H3K36me3 (Abcam 9050, GSM4977047), H3K4me1 (Abcam 8895, GSM4977051), H3K4me3 (Abcam 8580, GSM4977049) and H3K9me3 (ab8898, GSM4977045) ChIP-seq samples were used (28). Chromatin state discovery and characterization using all factors was carried out using ChromHMM (v1.23) (35).

For Hg38 ChromHMM generation the 65bp single-end reads (28) were trimmed to 54 bp reads and aligned to Hg38 using BWA (v0.7.17-r1188) to match newly generated ChIP sequencing data. H3K27me3 (GSM6429706), H3K36me3 (GSM6429700), H3K4me1 (GSM6429704), H3K4me3 (GSM6429702), H3K27ac (GSM6429695), H3K9me3 (GSM6429693) and H2A.Z (GSM6429697) reads were aligned to Hg38 using BWA (v0.7.17-r1188). Mapped reads were filtered for mapping quality using samtools (>20) (36). Duplicates were marked with Picard MarkDupes function (v2.18) (http://broadinstitute.github.io/picard/). Chromatin state discovery and characterization using all factors was carried out using ChromHMM (v1.23) (35). Read count data in 10kb bins across the genome and visualizing the resulting Pearson correlation heatmap was generated using deepTools (v2.0) computeMatrix and plotCorrelation functions (37).

### TIDE

TIDE (tracking of indels by decomposition) was used to estimate the editing efficiency of the crRNAs shown in Figure 5F (38). In short, editing efficiency was quantified by Sanger sequencing of the edited region with the primer sets shown in Supplementary Table S4. The PCR products were subjected to Sanger Sequencing and analyzed by the TIDE method. For the generation of the destroyed-target site cell lines, TIDE was used to pick clones without a wild-type sequence.

### PI profile

For cell cycle analysis, RPE-1 wild-type cells were plated in a six-well dish. The next day, the cells were transfected with crRNAs (see methods Transfections) and 24 hours later harvested in the. The cells were then fixed in cold 70% ethanol and kept at 4°C. Before staining, cells were pelleted and resuspended with 1:100 RNase (100μg/ml stock) and 1:1000 μl PI (from 50 μg/ml stock solution). Cells were recorded with the Attune NxT (Thermo Fisher Scientific) andFlowJo™ v10 Software (Biosciences) was used to analyze cell cycle distributions.

## RESULTS

### Probing location-dependent effects of a double-stranded break using CRISPR/Cas9

To systematically probe for location-specific toxicity of a DSB we performed CRISPR/Cas9 screens in which we induced individual DSBs across thousands of locations throughout the human genome. For this, we designed a sgRNA library that targets 6237 random genomic locations. These sgRNAs were designed such that potential off-target sites have at least two mismatches (see methods), to minimize off-target cutting and maximize induction of a DSB at a single location. We included 100 non-cutting sgRNAs (control) in the library which allow for quality control of the screens. This custom library covers all chromosomes and targets genic as well as non-genic loci (Supplementary Figure S1A, B). Using this custom sgRNA library we assessed DSB-toxicity in untransformed RPE-1 cells, as these cells have intact cell cycle checkpoints and are proficient for p53 (5,23,39). Given the established role of p53 in the response to a DSB (23), we also assessed DSB-toxicity in p53-deficient RPE-1 cells.

First, we determined the timing of DSB-induction using five random sgRNAs from our library, as the induction rate of Cas9-induced DSBs may differ depending on factors such as sgRNA design or chromatin compaction (40). We determined the accumulation of indels of these five individual sgRNAs by TIDE analysis (38) at 4, 8 and 16 days after sgRNA-infection. For sgRNAs SCC_4547, SCC_5988 and SCC_4107 all the target loci were edited at day 4 post-infection, whereas sgRNAs SSC_2344 and SSC_0765 only showed increased indel formation at the target sequence at later time points (Supplementary Figure S1C). Therefore, we decided to collect genomic DNA from the screens at three different time points (4, 8 and 16-day post-sgRNA-infection), to allow for the completion of Cas9-mediated DSB-induction for all target loci.

For the screen, cells were infected with the custom sgRNA library (Figure 1A), at a low multiplicity of infection (MOI) (Supplementary Figure S1D), and infected cells were selected with puromycin for 2 days. Then, cells were harvested on day 4 post-infection, to estimate representation of the library, and on days 8 and 16 post-infection, to determine sgRNA depletion over time. This enables us to uncover early- and late-editing events. Next, we measured the abundance of each sgRNA by next-generation sequencing. As expected, the abundance of control sgRNAs remained constant regardless of Cas9 expression or p53-status (Supplementary Figure S1E). For the p53-wildtype and p53-deficient screen we ranked the sgRNAs based on their mean fold-change in abundance. We found sgRNA hits in two categories; (I) sgRNAs that were less abundant at both time points and (II) sgRNAs that dropped out most at the 16-day time point (Figure 1B). This variation most likely reflects differences in either cutting efficiency by Cas9, DSB repair rate, or both. When comparing the mean fold-change in sgRNA abundance at 16-days in p53-wildtype and p53-deficient cells most of the DSB induced toxicity was abolished in the p53^-/-^ screen (padj < 0.05) (Figure 1C). This indicates that DSBs induced by these sgRNAs effectively suppressed cell proliferation in a p53-dependent manner, which is consistent with the established role of p53 in the DNA damage response (23,41). Taken together, our unbiased, genome-wide screening enabled us to identify a set of sgRNAs that produce a prominent growth inhibitory effect from a genome-wide library of 6237 single-cutting sgRNAs.

### Data-driven framework to design CRISPR/Cas9 crRNA pools targeting specific chromatin features

**Figure 2.**
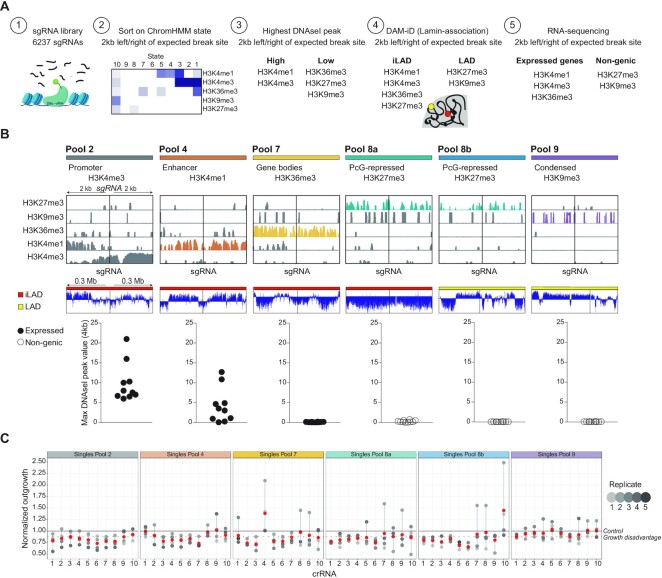
Data-driven framework to design CRISPR/Cas9 crRNA pools targeting specific chromatin features. (**A**) Strategy to computationally select 10 crRNAs located within the same chromatin environment. (**B**) Depiction of the six computationally computed crRNA pools. One representative crRNA of each pool showing the respective ChIP-seq signal 2 kb upstream and downstream of the DSB. LAD or iLAD signal is depicted for regions of 0.3Mb upstream and downstream of the DSB. Lastly, the graph on the bottom indicates the maximum DNAseI hypersensitive peak value within 2 kb upstream and downstream of the DSB for all crRNAs that belong to the indicated pool. (**C**) Cellular outgrowth 7 days after transfection of the corresponding single crRNAs into RPE-1 iCut cells. Outgrowth was measured and quantified using crystal violet (see methods). Mean (red) ± s.d. of at least three independent experiments.

In parallel to the screen, we used a second, independent method to identify location-specific differences in toxicity of a DSB. We aimed to design crRNA pools targeting specific chromatin features. For that, we had to determine (i) the target chromatin-types, (ii) the genomic locations of these types of chromatin in RPE-1 cells, (iii) filter the 6237 sgRNAs based on all these features (Figure 2A). To determine the chromatin landscape in RPE-1 cells, we selected five different histone marks, allowing for the identification of prominent chromatin types, and performed chromatin immunoprecipitation (ChIP)-seq experiments (Supplementary Figure S2A). This includes histone modification H3K27me3 (polycomb repressed) (42,43), H3K9me3 (heterochromatin) (44,45), H3K36me3 (gene bodies) (46), H3K4me1 (enhancers) (47), and H3K4me3 (promoters) (48) (Supplementary Figure S2B). Replicate ChIP-sequencing experiments were highly comparable and reproducible (Supplementary Figure S2B). These ChIP-sequencing data was combined to identify epigenetic patterns using ChromHMM. This algorithm used the 5 histone marks provided to define 10 distinct chromatin environments (Figure 2A2). To generate crRNA pools, we choose 4 ChromHMM states that are defined by the enrichment of only one specific histone mark each: state 2 - promoters (H3K4me3), state 4 - enhancers (H3K4me1), state 7 - gene bodies (H3K36me3) and state 8 - polycomb repressed (H3K27me3). A state defined by pure heterochromatin (H3K9me3) was unfortunately not present. Therefore, the selection crRNAs targeting H3K9me3 regions was based on high-coverage ChIP-sequencing from a publicly available RPE-1 dataset (49). We confirmed that all sites targeted by this pool of crRNAs are located in ChromHMM state 9 (pool 9, heterochromatin), a state with no enrichment for any of the other marks that were included in this dataset.

crRNAs of the pools are first selected based on the presence of the respective ChromHMM state in a 4 kb window around the break site. From this starting point, ten crRNAs were selected for each pool based on further characteristics (Figure 2A). DNAseI hypersensitivity data of the same cell line was used to define accessible regions, often used for identification of promoter and enhancer sites, which are known to encompass open chromatin regions to allow recruitment of transcription factors (32). The presence of a high DNAseI hypersensitive peak in a 4 kb window around the expected break site was assessed to select sgRNAs in states 2 (‘promoters’) and 4 (‘enhancers’), containing H3K4me1 and H3K4me3, respectively (Figure 2A3). Next, lamin-B1 DAM-ID data of RPE-1 cells was used to select sgRNAs from the pools in iLADs (interLADs) or LADs (lamin-associated) (Figure 2A4). Since it has been shown that reduced binding of HR proteins was observed at a locus that was artificially directed towards the lamina (50). Associated with this, a study pointed out that lamin A/C depletion leads to an impairment of base-excision repair (BER), pointing towards influences of lamin-association in repair (51). State 8 (‘polycomb repressed’), enriched for H3K27me3, was divided into two pools, sgRNAs in 8a targeting genomic loci in iLADs (‘polycomb repressed, iLAD’) and sgRNAs in 8b introducing breaks in LADs (‘polycomb repressed, LAD’), thereby assessing the contribution of lamina-association in the DSB-response. Lastly, to minimize toxic effects based on the location of the sgRNA target site in a gene exon, RNA-sequencing data was used to select for those sgRNAs that target either expressed, intronic regions or non-expressed, non-genic regions (Figure 2A5). Even though we selected crRNAs targeting regions outside exons, it is impossible to exclude small point mutations or larger deletions that could result in disrupted protein functions or potentially affect transcription by disrupting transcription-factor binding sites. These selection steps generated 6 highly uniformal pools of ten crRNAs each, one crRNA example of each pool is highlighted in Figure 2B. The sgRNAs from each pool were evenly distributed across the genome and each pool contained a maximum of two breaks per chromosome (Supplementary Figure S2C). To complement the data on sgRNA abundance of the screen, we transfected the 60 single sgRNAs from the pools using a non-viral transfection protocol (crRNA, see methods) in Cas9-expressing RPE-1 cells and 24h following transfection cells were replated to allow for 6 more days of growth. The target crRNA was hybridized with the transactivating CRISPR RNA (tracrRNA) and transfected into the host cell, which allowed for faster Cas9 cutting (52). The cellular response to these breaks was heterogeneous, ranging from minimal to severe inhibition of overall cell proliferation, indicating a difference in toxicity between crRNAs (Figure 2C). Taken together, these data imply that the location of a break could play a determinant role in the overall effect of the DNA damage response on cell proliferation.

### Location-independent effects of distinct sgRNAs

**Figure 3.**
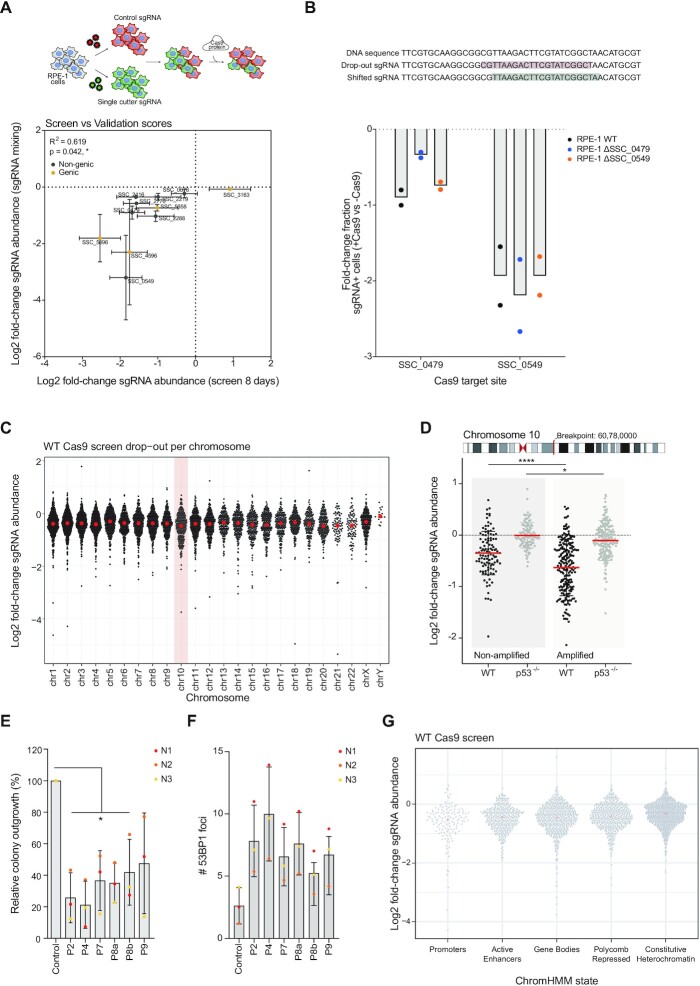
Toxicity of CRISPR/Cas9-mediated DSBs is mainly determined by the amount of DSBs induced, rather than the respective chromatin environment. (**A**) RPE-1 cells were infected with a non-targeting (control) sgRNA (co-expressing mCherry) or with a single cutter sgRNA (co-expressing sfGFP). After selection of sgRNA-infected cells, mCherry+ and sfGFP+ cells were mixed and infected with Cas9 virus or left untreated. mCherry:sfGFP ratios were calculated on day 0 and day 7. The fold-change in sgRNA abundance of this mixing experiment was compared to the fold-change of the corresponding sgRNAs on day 8 of the screen (Figure 1A,B). Mean ± s.d. (**B**) Off-target activity is measured in a mixing experiment similarly to (**A**). First, cell lines with individually mutated target sites were generated by transfection of crRNA:Cas9 complexes that create indels into the original target site (indicated on top). These mutated cell lines are then infected with control sgRNAs (co-expressing mCherry) and a single cutting sgRNA (co-expressing sfGFP). The fold-change of two independent replicates is plotted for two sgRNA hits from the screen ± s.d. (**C**) Fold-change of sgRNA abundance in wild-type Cas9-expressing RPE-1 cells, on day 16 of the screen, plotted per chromosome. (**D**) Fold-change of sgRNA abundance in wild-type and p53-deficient RPE-1 cells expressing Cas9, at 16 days of the screens, for all sgRNAs targeting chromosome 10. The amplification of a part of the q-ARM starts at chr10:60780000 (Hg19). (**E**) Quantification of the relative colony outgrowth 6 days after transfection with either of the 6 crRNA pools crRNAs that each contain 10 crRNAs (Figure C&D). Mean of three independent replicates ± s.d. Significance was calculated using an unpaired Student's *t*-test, * *P* ≤ 0.05. (**F**) Quantification of the number of 53BP1 foci 24h after crRNA transfection of pooled crRNAs. The mean ± s.d. was determined from three independent replicates. (**G**) Fold-change of sgRNA abundance in Cas9-expressing wild-type RPE-1 cells, on day 16 of the screens, separated based on the ChromHMM state of the target region (as determined in Figure 1C&D and see methods). The mean value is plotted in red.

To validate the hits from our genome-wide screen, we selected the 10 sgRNAs which inhibited cell proliferation to various degrees and targeted genic and non-genic regions (Figure 3A). We performed competition assays in which cells were either infected with a viral construct expressing a control (non-targeting) sgRNA co-expressing mCherry or a drop-out (targeting) sgRNA co-expressing sfGFP. These populations were mixed 1:1 and subsequently infected with a virus carrying Cas9. The mCherry:sfGFP ratio was determined one week later. Using this setup, we could confirm the screen results, where clear differences in DSB-toxicity were observed between locations. Furthermore, the results from this experiment show a significant correlation with the results obtained from the screen (*R*^2^ = 0,6193) (Figure 3A).

To rule out that the more toxic sgRNAs were detrimental for cellular fitness due to the induction of multiple DSBs, caused by possible off-target cleavage by Cas9, we set up an experiment to test whether off-target cutting caused toxicity. For the two most toxic, non-genic sgRNAs of the screen, SCC_0479 and SCC_0549, we designed two new crRNAs (shifted-crRNA) that would mutate the original target site of the sgRNA (see methods). Using these shifted crRNAs, we generated cell lines, RPE-1 ΔSCC_0479 and RPE- ΔSCC_0549, in which one of the original sgRNA sequences is mutated (Supplementary Figure S3A). Next, we determined the outgrowth deficiency of the two most toxic sgRNAs from our screen in the corresponding target-mutated cell lines, using the same mCherry-sfGFP mixing set-up as described before. If the outgrowth deficiency of these two sgRNAs is due to on-target DSB-induction, mutation of the target site should lead to normal proliferation. However, if mutation of the target site does not or only partially rescue outgrowth deficiency, this would indicate that the toxic effect is most likely due to off-target DSB-induction. We find that SSC_0479 target site mutation reduced the cell proliferation induced by sgRNA SCC_0479 by ∼50%, indicating that the toxicity induced by this sgRNA is partially due to off-target DSB-induction. However, for sgRNA SCC_0549, the off-target effect appeared to be more drastic, as sgRNA SCC_0549 was equally toxic in wild-type cells and cells lacking the original target sequence (Figure 3B). Thus, differential inhibition of cell proliferation by the most toxic sgRNAs from our screen can be (partially) attributed to off-target DSB-induction.

Our genome-wide screen allowed us to identify a set of sgRNAs that are more detrimental to cell proliferation than average. To rule out that breaks in specific chromosomes are more detrimental as compared to others, we plotted the drop-out rates per chromosome. No clear differences were observed between breaks across all chromosomes and we find that the effects are largely p53-dependent (Figure 3C, Supplementary Figure S3B). This implies that various sgRNAs cause a different amplitude of damage response activation. This could either be dependent on the chromatin context in which the break occurs or the difference could be due to variable cutting efficiencies or off-target editing.

To independently assess if our screen identified dose-dependent toxicities, we analyzed the depletion of sgRNAs targeting chromosome 10. For this, it is important to note that RPE-1 cells contain a trisomic region of chromosome 10. When assessing the differences in outgrowth between sgRNAs targeting the non-amplified versus the trisomic region of chromosome 10, we found a significant difference between the two populations (Figure 3D), indicating that the amount of DSBs correlate with cellular toxicity, which we observe to be partially rescued in p53^–/–^ (Figure 3D). This indicates that a dose-dependent DNA damage-induced arrest. In contrast, a context-dependent effect is not obvious from these data.

Next, we turned to our biased approach to test the effect of chromatin context on the damage response. At first, 10 single crRNAs targeting chromatin states 2, 4, 7, 8a, 8b, and 9 were combined into six pools, each targeting a given chromatin state. Subsequently, we determined the toxicity of DSBs in different chromatin states by transfecting these pools and assessing cellular outgrowth by colony-forming assays. As expected, the induction of 10 DSBs severely reduced cellular outgrowth, to a much larger extent than the introduction of a single DSB (Figures 2C and 3E), another implication that dose is a much more important determinant of outcome than chromatin context. Indeed, the extent of DSB-induced toxicity was independent of chromatin state, although 10 DSBs in chromatin states 2 (‘promoters’) and 4 (‘enhancers’) appeared to be slightly more toxic (Figure 3E). To assess whether the pools have similar DSB-induction, we characterized the break induction potential of each pool of crRNAs by measuring the number of 53BP1 foci, a DSB marker (53), 24h after crRNA pool transfection. Clear 53BP1 foci induction was observed when pooled crRNAs were transfected compared to a non-targeting control sgRNA (Figure 3F, Supplementary Figure S3D). Importantly, DSB-induction was most efficient when targeting chromatin state 4 (‘enhancers’, ten 53BP1 foci) and least efficient in chromatin state 8b (‘polycomb repressed, LADs’, five 53BP1 foci), consistent with the observed differential toxicity. This is most likely a reflection of the differential accessibility of these sites, with the chromatin state 4 (‘enhancers’) crRNAs targeting open chromatin, versus the chromatin state pool 8b (‘polycomb repressed, LADs’) targeting heterochromatin (40). This is in line with our previous observation, where a higher number of DSBs are associated with more dramatic outgrowth defects (Figure 3D). Altogether, these data show that the chromatin-dependent effects on DSB-toxicity, if present, are very subtle, whereas the number of breaks induced plays a much more determinant role.

### Single DSBs can efficiently pause the cell cycle independent of chromatin context

The data presented so far show clear differences in toxicity of 60 individual Cas9-induced DSBs (Figure 2C). Yet, the influence of chromatin on DSB-toxicity seems subtle (Figure 3E, F, G). We hypothesized that this limited chromatin effect could be explained by incomplete characterization of the chromatin context. Therefore, we extended our chromatin classification through the characterization of two additional histone marks. Histone mark H2A.Z was added since it has been shown to be required for the loading of the BRCA1 complex and it is exchanged at DSBs to convert the chromatin to an open conformation (54). Additionally, H3K27Ac was added to distinguish active enhancers (H3K27Ac) from inactive/poised enhancers (H3K4me1) (55). We performed ChIP-sequencing for H3K9me3 (heterochromatin), to replace the original H3K9me3 ChIP, H2A.Z (active transcription) (54), and H3K27Ac (active enhancer), and re-defined chromatin states (by ChromHMM), including these additional histone marks (Supplementary Figure S4A–C). To verify that the newly computed chromatin states robustly classify the crRNAs to the equivalent state, we repeated the systematic classification as discussed in Figure 2A. Comparison of the chromatin states targeted by our set of 60 crRNAs as determined by our initial and updated ChromHMM-analysis demonstrated that only 4 of the 60 crRNAs target sites (2.2, 2.9, 4.6 and 8b.4) had an altered chromatin state classification (Supplementary Figure S4D). Thus, including these additional histone marks enhanced the overall identification of chromatin states, allowing us to make clear predictions of the chromatin state in which each DSB is generated. As most chromatin states targeted by our crRNAs were accurately predicted by our initial chromatin state definition, we are more confident with the characterization of the DSB-chromatin context. Therefore, this analysis confirms our previous chromatin classification and thereby reinforces our hypothesis that the chromatin context is unlikely a confounding factor for Cas9-dependent DSB-toxicity.

**Figure 4.**
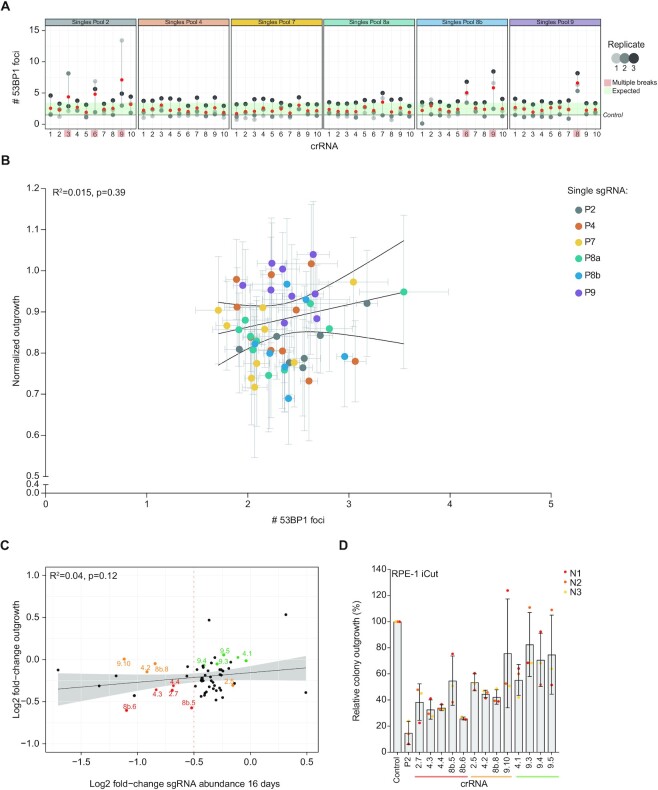
Heterogeneity in toxicity after transfection of different single cutting crRNAs. (**A**) Quantification of the number of 53BP1 foci per cell, 24h after transfection of individual crRNAs from all six pools. Based on these results, we consider crRNAs to induce off-target DSBs in case the number of 53BP1 foci in each replicate exceeds the boundaries of the s.d. within the experiment. Mean (red) ± s.d. of three independent experiments. (**B**) Correlation plot between normalized outgrowth and the number of 53BP1 foci after crRNA transfection. Both measurements were performed within the same transfection (Mean ± s.d. of at least three independent experiments). (**C**) Correlation plot comparing the fold-change of sgRNA abundance on day 16 in the screen and the fold-change in outgrowth of RPE-1 cells transfected with each of the 60 single crRNAs (Figure 1E). Color-coded by the reduction in outgrowth: severe reduction in outgrowth in both approaches (red), reduction in outgrowth in one of the two (orange), no effect on outgrowth (green). (**D**) Relative colony outgrowth of RPE-1 iCut cells after transfection with single cutting crRNAs selected from (**C**). Mean ± s.d. of three independent experiments.

After verifying the chromatin classification of the crRNAs, we sought to exclude the observed differences in DSB-toxicity to be dose-dependent. As an increasing amount of DSBs results in more toxicity (5,23), it raises the question of whether the heterogeneity in toxicity between sgRNAs is a result of multiple DSBs induced by one sgRNA. This is a possibility, as it has been shown that Cas9 is prone to induce multiple DSBs because of off-target editing. To evaluate if dose-dependent DSB-toxicity could be explained by off-target editing by Cas9, we stained for 53BP1, a canonical DNA damage marker (53), in Cas9-expressing RPE-1 cells 24 hours after crRNA transfection. We find that most of the crRNAs induce 1–3 53BP1 foci (Figure 4A), which fits with a predicted number of target sites, and indicates that these crRNAs only induce on-target DSBs. However, 5 out of the 60 crRNAs (i.e. 2.6, 2.9, 8a.7, 8b.6, 9.8) show higher amounts of 53BP1 foci (4–7 breaks on average), implicating off-target DSB-induction events. For further analysis, four crRNAs (2.6, 2.9, 8a.7 and 9.8) were excluded, while crRNA 8b.6 was taken along as a positive control for off-target DSB-induction. Comparison of cellular outgrowth to the number of 53BP1 foci that were induced by on-target crRNAs showed no correlation between outgrowth and DSB-induction (*R*^2^ = 0.015, Figure 4B). Furthermore, the patterns of normalized outgrowth and break induction (53BP1 foci) of the crRNAs of each individual chromatin environment appeared to be heterogeneous. It seems, that if present, the effects of chromatin context on Cas9-induced DSBs are minor.

Since the observed differences in DSB-toxicity after crRNA transfection are small, we combined outgrowth data of the two parallel approaches from the same sgRNAs to find common toxic DSB-sites (Figure 4C). Unfortunately, no clear correlation was observed between the two parallel approaches, but several sgRNAs displayed relatively high toxicity in both settings (marked in red). We hypothesized that truly toxic DSB-sites will be identified both in a lentiviral system (as was the case in the screen), and an RNA-based transfection approach. We picked 13 locations that were of interest because of their similar impact on growth, irrespective of the method of crRNA/sgRNA delivery (Figure 4C). The chosen DSB-locations were divided into three categories: (i) locations that cause a severe defect in cellular outgrowth independent of the transfection method, (ii) locations showing intermediate cellular outgrowth defects in the screen, or (iii) locations that did not show any proliferative disadvantage after break induction. Since the observed outgrowth defect after DSB-induction between the screen and our validation with crRNAs was heterogeneous, we confirmed DSB-toxicity in an additional experiment. For this, Cas9-expressing RPE-1 cells were transfected with crRNAs and 24 hours later re-plated for a colony-forming assay. We find a minor decrease (∼10–20%) in colony outgrowth compared to control crRNA transfected cells after DSB-induction by transfection with crRNA 9.10/4.1/9.3/9.4/9.5, confirming that DSB-induction using these sgRNAs is not enough to cause a significant proliferative disadvantage (Figure 4D). DSB-induction using more toxic crRNAs, 2.7/4.3/4.4/8b.5/8b.6/8b.8, showed a major decrease in colony outgrowth (∼60–80%) compared to control crRNA-transfected cells. In parallel, cells were fixed and stained for damage-induced 53BP1 foci, confirming the same number of foci as measured before (1 to 3 53BP1 foci, Supplementary Figure S4E). This data is consistent with the previous classification based on the correlation between the results from the screen and crRNA outgrowth experiments (Figure 4D). We conclude that we could identify DSB-locations that cause severe, intermediate and no proliferative disadvantage after break induction, indicating that there are differences in toxicity after CRISPR/Cas9-mediated DSB-induction.

Since we observed that 10 DSBs resulted in a similar short-term arrest independent of the chromatin state (Supplementary Figure S3F), we investigated whether cells arrest in a specific cell cycle phase after encountering one Cas9-induced DSB and if that is affected by specific chromatin features surrounding the break site. Therefore, we set out to monitor cell cycle distribution 24h after crRNA transfection. We confirmed that cells accumulate in G2 after break induction by analysis of DNA content, as evidenced by an increase in cells with a 4N DNA content (Supplementary Figure S4F). This effect is similar across different crRNAs and independent of the chromatin state. Together, we observe that DNA damage signaling is activated by Cas9-induced DSBs and that targeting a single site in the genome is sufficient to elicit a checkpoint response (23). This signaling is enough to halt in a 4N phase of the cell cycle, indicating that cells can respond to very low levels of genotoxic stress, but cope with this without removing themselves from the proliferating population.

### Toxicity of single cutting crRNAs correlates with cutting efficiency

**Figure 5.**
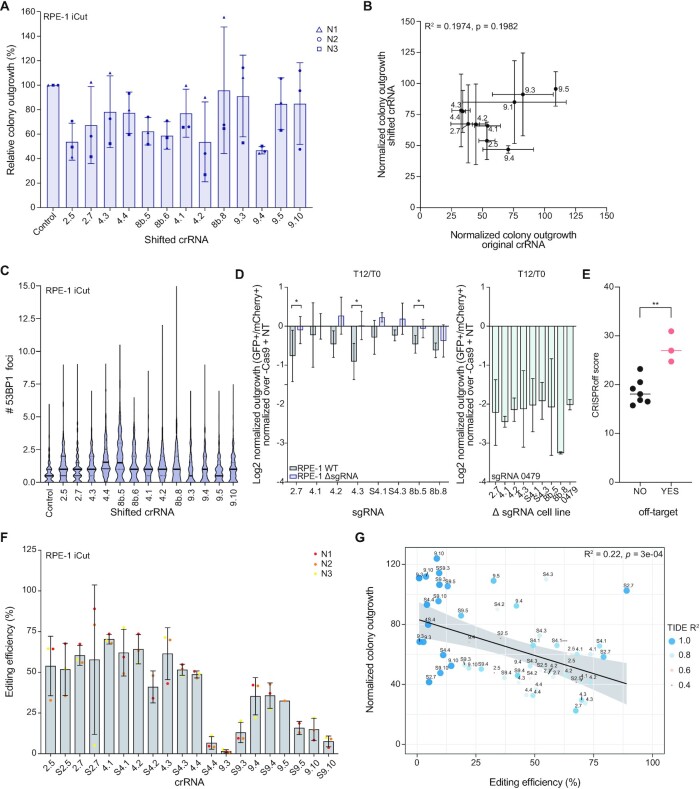
The toxicity of a CRISPR/Cas9 mediated DSB is mainly determined by Cas9 cutting efficiency. (**A**) Relative colony outgrowth of RPE-1 iCut cells 7 days after transfection with the shifted single cutting crRNA. Mean ± s.d. of three independent experiments. (**B**) Pearson correlation between the normalized colony outgrowth of the original crRNA (depicted in Figure 1E) and the corresponding shifted crRNA as measured in (A). (**C**) Quantification of the number of 53BP1 foci for the shifted single cutting crRNA 24h after crRNA transfection. (**D**) WT RPE-1 and RPE-1 cells in which individual sgRNA targets were mutated, were infected with a control sgRNA (NT, co-expressing mCherry) or a single cutter sgRNA (co-expressing sfGFP) and mixed 1:1, allowing for off-target cutting evaluation (WT and mutated cells with single cutting sgRNAs on the left, mutated cells with sgRNA SSC_0479 on the right). mCherry and sfGFP ratios were assessed before and 12 days after Cas9 expression. The relative ratio, normalized over Cas9- cells is plotted. The mean of three independent experiments is shown ± s.d. (**E**) CRISPRoff score (indicating the efficiency of editing the predicted off-target sites) was plotted for all sgRNAs that were used for two independent off-target assessment experiments (Figures 3B and 5D). Significance was calculated using an unpaired Student *t*-test, ** p ≤ 0.05. (**F**) Editing efficiency of RPE-1 iCut cells after transfection with single cutting crRNAs was determined by TIDE. Depicted are three independent replicates ± s.d. (**G**) Correlation plot comparing the colony outgrowth after transfection of single crRNAs and the corresponding editing efficiency as measured in (F). The analysis is performed using a linear regression model.

To investigate if the proliferation defect after a crRNA transfection is a result of a single DSB, we mutated the sites targeted by our crRNAs. For this purpose, we designed a shifted crRNA for each target-location crRNA, such that the shifted crRNA would mutate the original crRNA target sequence. To be able to achieve that, a sgRNA within the original crRNA sequence containing an NGG PAM should be available to have on-target activity (56). This design was possible for 8 out of the 13 chosen crRNAs, due to limitations in the available PAM sequences. Since these shifted crRNAs target the same chromatin features as their original counterparts, we hypothesized that the toxicity after break induction should be similar. As expected, a decrease in relative colony outgrowth was observed after DSB-induction with the shifted crRNAs (Figure 5A). However, we observed no correlation between the outgrowth after transfection of the original crRNA and its shifted counterpart (Figure 5B). This again implies that using CRISPR/Cas9, we find no evidence of chromatin context or location of a DSB as determinants of DSB-toxicity. To determine whether off-target DSB-induction was responsible for the poor correlation between original and shifted crRNAs, we assessed the amount of DSBs that was induced by transfection of the shifted crRNA and found induction of 1–2 average breaks by analysis of 53BP1 foci, similar to its original counterpart (Figure 5C, Supplementary Figure S4E).

We previously selected different sgRNAs based on the outgrowth defects they induced in the screen and our validation experiment (Figure 4C). To rule out that the more toxic sgRNAs are not inducing a stronger outgrowth deficit due to induction of multiple DSBs, we set up an experiment to test whether these sgRNAs are causing off-target cutting. Using the eight shifted crRNAs, we generated eight cell lines in which individual target sites are destroyed (i.e. ΔSSC#), for which we performed sgRNA mixing experiments. Next, we determined the toxicity of all those eight sgRNAs in wild-type cells and the corresponding target-mutated (ΔSSC) cell lines, using the same mCherry-sfGFP mixing set-up as described before. We find that mutating the target site of seven out of the eight sgRNAs alleviated the toxicity induced by its sgRNA completely, indicating that the toxicity induced by those sgRNAs is caused solely by on-target DSB-induction. However, for one sgRNA (8b.8), mutation of the target site did not significantly reduce the toxicity induced by sgRNA 8b.8, indicating that this sgRNA induces off-target DSBs that cause the toxicity of this sgRNA (Figure 5D). Thus, differential inhibition of cell proliferation by the most toxic sgRNAs is caused by on-target effects.

The accuracy of our determination of off-target inducing sgRNAs was confirmed by a predictive algorithm, CRISPRoff (v1.2, https://rth.dk/resources/crispr/crisproff/ (57)). For each sgRNA from our validation experiments the CRISPRoff value was computed (Figures 3B and 5D). The sgRNAs were classified into either having detected off-targets (YES) or not showing off-target cutting in our experiments (NO). When comparing the two groups, we find a significantly increased CRISPRoff value for sgRNAs that showed off-target cutting (Figure 5E). This indicates that using such prediction tool can help select sgRNAs with lower off-target cutting efficiency and are of importance when designing sgRNAs for future experiments.

As an alternative explanation for differential inhibition of cell proliferation by the various sgRNA/crRNAs, we considered that difference in editing efficiency could play a decisive role. To test this, we determined the editing efficiency of each individual crRNA by measuring the accumulation of indels by TIDE (38), at 72h after crRNA transfection and Cas9-mediated DSB-induction and repair. Strikingly, the editing efficiency of the crRNAs was highly variable, ranging from very low, 2–10% cutting efficiency (S4.4, 9.3, S9.5, 9.10 and S9.10) to 60% cutting efficiency (2.5, S2.5, 2.7, 4.1, S4.1, etc.) (Figure 5F). Interestingly, except for crRNA S4.4, all crRNAs that show reduced indel frequencies are located in H3K9me3 regions. On average, crRNAs targeting more closed chromatin (Pool 9, H3K9me3) showed lower indel frequencies than crRNAs targeting open chromatin (Pool 4, H3K4me1). Note that, within heterochromatin, the magnitude of this effect varied depending on the crRNA. These results indicate that the overall indel frequency depends on the local chromatin context.

An important remaining question is whether differential Cas9-editing efficiencies across locations could explain the DSB-toxicity. Comparison of Cas9-editing efficiency and outgrowth after crRNA transfections yielded overall indel frequencies that strongly correlated with the normalized outgrowth for each of the crRNAs (Figure 5G). Though, there are some limitations by using TIDE as an estimation for editing efficiency. First, with TIDE we are only able to measure on-target editing efficiency. As shown and highlighted in Figure 5D and E, some crRNAs create DSBs at off-target sites, which we are unable to measure. Also, TIDE uses a confidence measurement (*R*^2^), which demonstrates the reliance of Indel assessment. In Figure 5G, we show the differences observed in *R*^2^ in our experiments, which limit the precision of our TIDE measurements in Figure 5F. Furthermore, any DSB-repair event that restores the original sequence will not be detected by TIDE (i.e. repair through HR), leading to a potential underestimation of cutting events. Such restored sgRNA target sites could allow for re-cutting by Cas9, a potentially toxic event. We conclude that the toxicity of DSBs generated by CRISPR/Cas9 is a multi-step process, that requires reliable data from different layers to come to a concise conclusion.

Taken together, our analysis of context-dependency on DSB-toxicity shows that the effect of the local chromatin environment has limited influence on toxicity after a CRISPR/Cas9 mediated DSB. In contrast, we propose that the CRISPR/Cas9-associated effects of off-target cutting and editing efficiency are both leading to higher numbers of DSBs, thereby outcompeting any potential chromatin effects on DSB-toxicity.

## DISCUSSION

DNA lesions are commonly occurring throughout the entire genome. The chromatin context surrounding a DNA lesion has previously been shown to influence the repair pathway (16,58), but whether the location affects the toxicity of a break remained unresolved. Here we used high throughput screening of Cas9-induced DNA breaks to investigate if chromatin context affects the toxicity of a DSB. Using a custom-designed library of 6237 single-cutting sgRNAs, we found heterogeneity in the toxicity between sgRNAs. Enhanced toxicity of single sgRNAs in the screen could potentially come from editing essential genes or regulatory regions. Due to the large number of sgRNAs in the screen, we expect to level out this effect. Interestingly, it is possible that the slightly increased toxicity of sgRNAs targeting more open chromatin regions could be partially due to the preferred localization of regulatory elements like promoters and enhancers. Generating DSBs in such locations could be more detrimental to cell proliferation compared to other regions because the likelihood of disrupting a genic function after non-fateful repair of a DSB, which could cause toxicity.

After transfection with synthetic crRNAs, we find that cells either arrest in G1 or in a 4N-like state dependent on the break, and we have previously shown that a difference in cell cycle stage at the moment of DSB occurrence can explain this difference (6,7). In addition, we find that when cells lack p53, most of the cell fate decisions were abrogated when compared to cells proficient for p53. This is in line with previous work, where it has been shown that cell fate is dictated by the p53 activation during the DNA damage response (5,39). However, no correlation was observed between chromatin context and outgrowth deficiency. Instead, we find that CRISPR/Cas9 editing efficiency and off-target editing are the main determinants of the extent of the growth arrest. Consistently, we show that pools of crRNAs targeting the same chromatin context induce a growth delay that seems to correlate with their respective editing efficiencies. This emphasizes that the inherent limitations of CRISPR/Cas9 hinder its use as a tool to study the relation that a DNA break might have on the fitness of cells.

To determine the exact reason for differences between crRNAs in toxicity after a DSB, our high throughput analysis allows to monitor the impact of local chromatin on Cas9-editing. Differences in editing efficiency of Cas9 have been noticed before in multiple heterochromatin types, where the Cas9 editing is generally lower compared with euchromatin (16,59–62). A recent study shows that the local search ability of Cas9 is the most prominent distinguishing factor in determining editing outcomes at heterochromatin loci (63). Most likely the relatively low accessibility of the DNA and lower search interactions in these loci prevents efficient editing by Cas9 (63). Daer et al observed reduced editing efficiency associated with heterochromatin (closed state) due to a reduction in Cas9 binding. Here, we specify regions that are more open and marked with H3K4me1/H3K4me3 are most likely to be edited effectively and thereby also more likely to affect cell fate. In accordance, we find that cell proliferation was not hampered in cells encountering a DSB in DNA marked with H3K9me3 explained by lower editing efficiency. These findings further emphasize how these structural differences observed previously can explain the toxicity effects we observe in this study (59).

The nature of ‘open’-chromatin having higher editing efficiency could potentially lead to more off-targets located in these regions since these locations are more likely to be easily found and targeted by Cas9. Most of the off-target breaks, which were hallmarked by an increase in 53BP1 DNA damage foci, were associated with the sgRNAs from Pool 2 (‘promoters’), located in open chromatin marked with H3K4me3. Hypothetically, if the on-target site is broken effectively and potential off-target sites are edited simultaneously, multiple breaks at or around the same time increase our ability to visualize multiple DSBs by counting 53BP1 foci. We can observe this in the present study, as more off-target sites lead to quantifiably more toxicity after a DSB, pointing towards dose-dependent DSB-toxicity. The same holds true for the experiments where 10 crRNAs are combined to induce multiple DSBs targeting the same chromatin environment. First, the combination of multiple crRNAs, and therefore the induction of multiple DSBs in the same cell, could induce strong toxicity which potentially masks subtle chromatin state-dependent effects. Another limitation is that we limited the number of crRNAs per pool to 10 for this approach. Considering the size of the human genome, we could potentially miss effects due to limitations in the number of crRNAs used. Lastly, some pools contain crRNAs targeting the same chromosome. This could cause large-scale deletions, causing severe toxicity in those cells.

Even though the sgRNAs from the library were designed with at least two mismatches, we observed many of these off-target DSBs. When focusing on the combined results of the screen and the biased approach using the pooled crRNAs designed to target specific chromatin environments, we were able to find sgRNAs with similar DSB-toxicity. Unfortunately, no clear correlation was observed between the screen and the biased experiment of the pools, but this might be explained by the differences in the set-up of the experiment: in the screen, lentiviral plasmids were used to express the sgRNA, while in the biased approach synthetic crRNAs were used. Additionally, with many locations targeted in the screen, we could reliably demonstrate the dose-dependent on-target effects of Cas9, where targeting an amplified region inhibited outgrowth. This is in line with previous studies where targeting amplified regions in the genome harmed proliferation (64,65). Even though we tried to minimize off-target editing and control for it, we show that off-targets were nevertheless influencing our experiments. This could be explained by the fact that we use excessive amounts of Cas9/crRNA complexes which have been shown to tolerate mismatches in the guide matching region, ultimately leading to Cas9 editing (66). However, Fu et al showed that reducing the amount of sgRNA and Cas9 complexes in the cell was not sufficient to reduce off-target editing (67). Therefore, we propose that further optimization of the CRISPR/Cas9 system is necessary to perform reliable genome-wide assessments of DSB-toxicity by reducing off-target effects.

An important, not-addressed issue, is the role of the DNA sequence in Cas9-dependent editing. Several studies have profiled the role of the target DNA sequence in Cas9-dependent DNA repair outcomes where the repair outcomes are determined by the target sgRNA sequence rather than the genomic context site (68,69). Since target sequence could be linked to repair outcomes it is possible that the differences in outgrowth after DNA damage may as well be explained by the sequence of the sgRNA. In the future, it will be interesting to explore whether this could be a determinant of cell fate after CRISPR/Cas9 induced DSBs. Even though we aimed to cover the entire genome, repetitive sequences like centromeres, rDNA and telomeres cannot be assessed using CRISPR/Cas9 breaks. However, it would be interesting to study the toxicity of such breaks in light of the possible recombination events in such genomic context, as it is likely that toxic recombination events occur (21).

For clinical purposes, Cas9 is an increasingly sought-after tool for the treatment of various genetic diseases. In light of the results described here, it would be highly recommended to first verify the chromatin context of the sgRNA. Activating or targeting inactive genes -locations surrounded by compacted chromatin- is likely to be more difficult when using Cas9. Recently, single-molecule imaging of editing proteins reveals that TALEN is fivefold more efficient in heterochromatin as compared to Cas9, because Cas9 becomes encumbered by local searches on non-specific sites in these regions (63). If the use of Cas9 is necessary, modifying an already active gene is preferred, since they are often marked with active chromatin marks and contain multiple DNAseI-hypersensitive peaks, allowing for higher editing efficiency. Next, when targeting active genes, off-target cutting events need to be prevented, as this would lead to unintended editing of other regions within the genome.

The results obtained in this study have practical implications for genome editing using Cas9. It brings advantages such as immense targeting flexibility compared to y-irradiation and allows large scale screenings with more ease than other targeted genome editing strategies like TALEN. A caveat of Cas9 as a tool to induce DSBs is that Cas9 tends to stick to the break-ends after cutting (70). In line, end-resection of Cas9-induced DSBs appears relatively uni-directional (71). This leads to differences in the topology of the DSBs, not resembling *y*-radiation or V(D)J recombinase-induced breaks. Therefore, Cas9-induced DSBs can affect checkpoint signaling differently and these findings should not be extrapolated to IR-induced DSBs. Overall, results obtained in this study highlight distinct CRISPR/Cas9-mediated DSB effects and therefore serve as a guidance to engineer locations in cell culture or facilitate the use of CRISPR/Cas9 for therapeutic purposes.

## DATA AVAILABILITY

- The iKRUNC package was used to select sgRNA with a single unique target in the human genome (http://github.com/Toverkwark/ikrunc, previously described by Evers et al. (25))
- DESeq2 1.31.3 (R 4.0.5) (https://bioconductor.org/packages/release/bioc/html/DESeq2.html)
- Analysis pipeline in R, available from Github (https://github.com/eskoeleman/gRNA_environment)
- RefSeq data was acquired from NCBI (https://www.ncbi.nlm.nih.gov/refseq/)
- ImageJ software (NIH) (https://imagej.nih.gov/ij/download.html)
- Genome browser snapshots, heatmaps and density plots were generated using EaSeq (http://easeq.net)

- BWA (v0.7.17-r1188) (https://github.com/lh3/bwa/releases)
- Duplicates were marked with Picard MarkDupes function (v2.18) (http://broadinstitute.github.io/picard/)

- Pearson correlation heatmap was generated using deepTools (v2.0) (https://deeptools.readthedocs.io/en/develop/content/installation.html)

- Chromatin state discovery and characterization using all factors was carried out using ChromHMM (v1.23) (https://github.com/jernst98/ChromHMM/releases)
- TIDE (Tracking of Indels by Decomposition) was used to estimate the editing efficiency of the crRNAs (http://shinyapps.datacurators.nl/tide/)
- FlowJo™ v10 Software (Biosciences) (https://www.flowjo.com/solutions/flowjo/downloads)

- H3K9me3 ChIP-analysis dataset from GEO (GSM3105086); H3K36me3, H3K4me1 and H3K4me3, H3K9me3 dataset from GEO (GSE163315)
- Newly generated H3K9me3, H3K27Ac and H2A.Z ChIP are deposited in GEO (GSE210402)
- Data from GSE163315 (H3K36me3, H3K4me1 and H3K4me3) and ChIP-sequencing data from H3K27me3 was aligned to Hg38 for ChromHMM analysis and deposited in GEO (GSE210402)
- RNA-sequencing data of RPE-1 wild-type cells was previously performed in the lab and the dataset from GEO was used (GSE163315)
- Lamin-B1 DAM-iD data was retrieved from the 4DNucleome consortium (accession number: 4DNESHGTQ73M)
- DNaseI data from ENCODE (dataset: ENCSR000EON, file: ENCFF128BPC)
- ChIP-sequencing was previously performed in the lab and the dataset can be found at GEO (GSE163315)

## Supplementary Material

gkac758_Supplemental_Files

## Notes

Present address: Lenno Krenning, Dutch Cancer Society, 1062 EA Amsterdam, The Netherlands.

Present address: Emma S. Koeleman, Chromatin Networks, German Cancer Research Center (DKFZ), 69120 Heidelberg, Germany.

Present address: Jeroen van den Berg, Oncode Institute, Hubrecht Institute-KNAW (Royal Netherlands Academy of Arts and Sciences) and University Medical Center Utrecht, 3584 CT Utrecht, The Netherlands.

Present address: Bastiaan Evers, Scenic Biotech, 1098 XG Amsterdam, The Netherlands.

Present address: Anna G. Manjón, Children's Medical Research Institute, 214 Hawkesbury Road, Westmead NSW 2145, Australia.

Present address: Stacey Joosten, Novartis Pharma Nederland, 1101 BX Amsterdam, The Netherlands.

Present address: Yongsoo Kim, Department of Pathology, Cancer Center Amsterdam, Amsterdam UMC, Vrije Universiteit Amsterdam, 1081 HV Amsterdam, The Netherlands.
