# Supplementary material for: Double-strand break toxicity is chromatin context independent
Source: Nucleic Acids Res. 2022 Sep 15;50(17):9930–47. doi: 10.1093/nar/gkac758 (PMC9508844; doi:10.1093/nar/gkac758)
Supplement: gkac758_Supplemental_Files [file gkac758_supplemental_files.zip › Supplement figures and legends.pdf]

Suppl. Figure 1

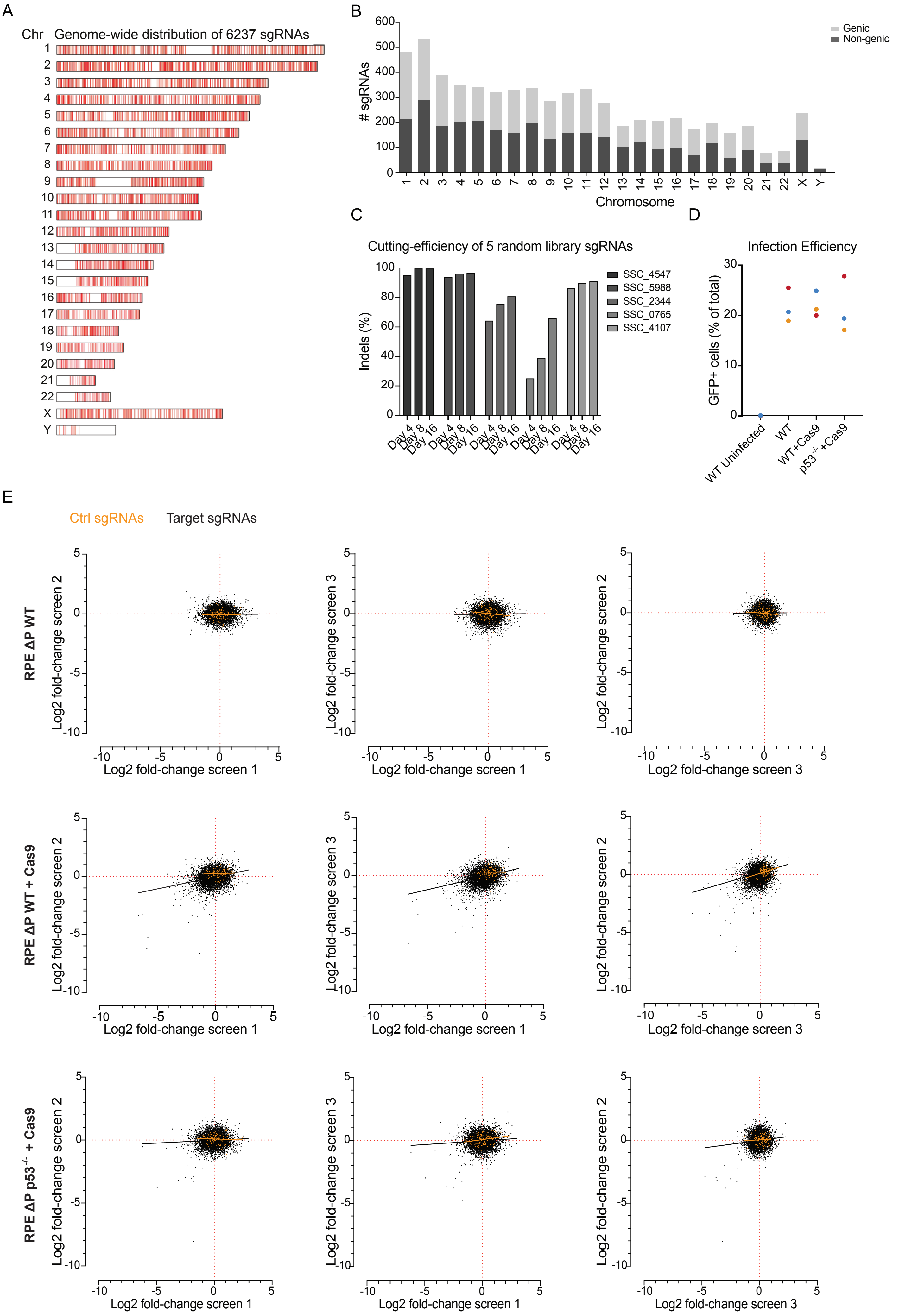

Supplement figure 1

A) An ideogram showing all locations targeted by the 6237 sgRNAs in our library that covers the full human genome. B) Overview of the number of sgRNAs targeting non-genic or genic loci per chromosome. C) The percentage of insertions and deletions in wild-type RPE-1 Cas9 cells measured by TIDE 4, 8, and 16 days post-infection of 5 randomly selected sgRNAs of the CRISPR/Cas9 single cutter library. D) Infection efficiency of the CRISPR/Cas9 Single Cutter library in different arms of the single cutter screen, as determined by the percentage of sfGFP+ cells. E) Correlation plots comparing the fold-change of sgRNA abundance in the three screen replicates in different cell lines. All the targeting sgRNAs are in black and the 100-control sgRNAs are in orange.

Suppl. Figure 2

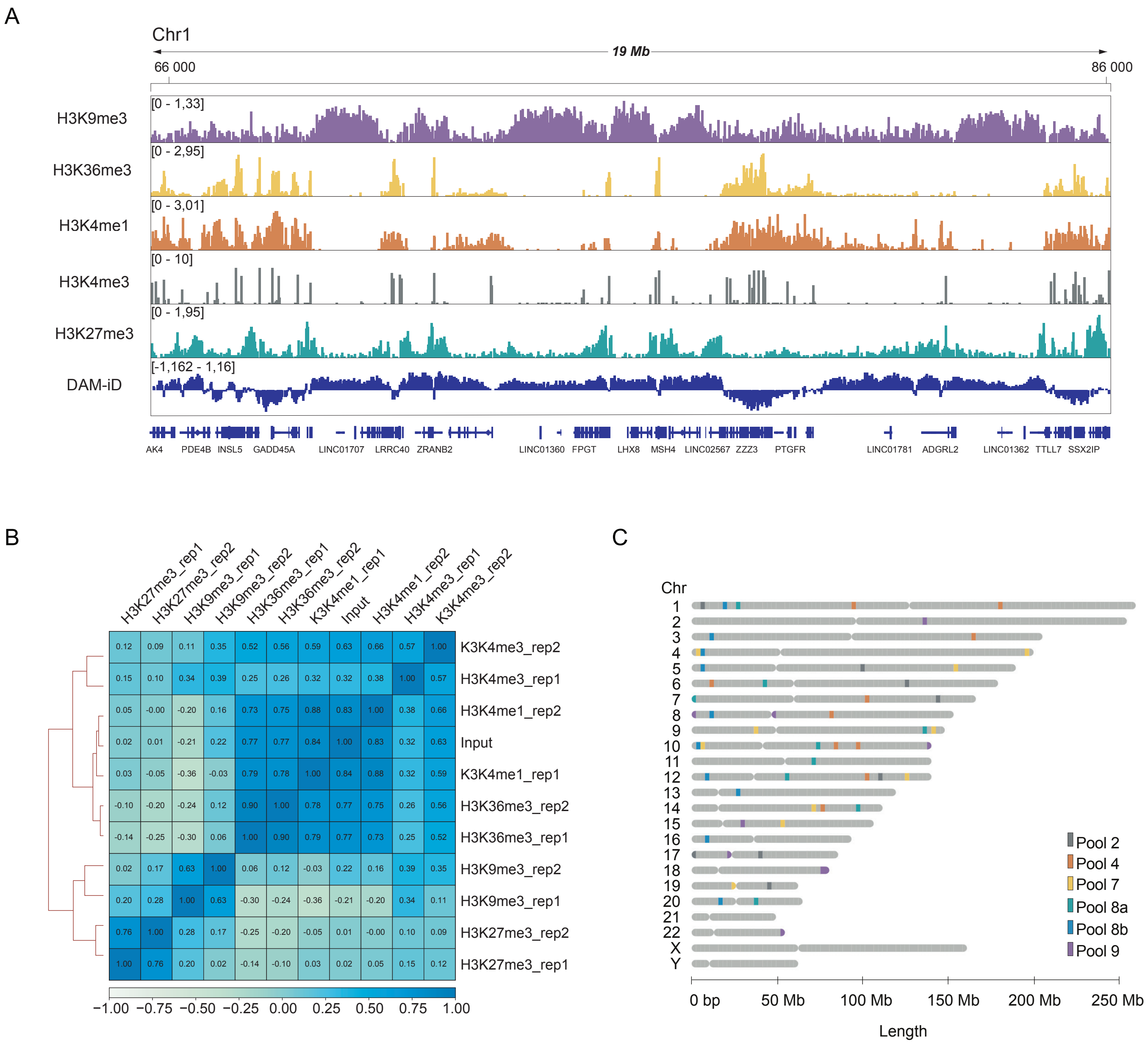

Supplement figure 2

A) Representative plot of one replicate of H3K9me3, H3K36me3, H3K4me1, H3K4me3, and H3K27me3 ChIP-sequencing tracks from the p-ARM of chromosome 1 in RPE-1 iCut cells. Reads were aligned to Hg19. DAM-ID track shows nuclear lamina (NL) interactions in RPE-1 cells that were determined by LaminB1-DamID (29, 30). Positive values indicate enrichment in NL interactions negative values indicate NL detachment, relative to DAM-only control (see methods). B) Correlation of the read-counts (10kb bins across the genome) of two independent replicates of ChIP-sequencing of the indicated histone-marks aligned to Hg19. C) Ideogram showing the locations that are targeted by any of the 10 crRNAs that belong to the 6 pools that were created using chromoMap (71). Locations were mapped based on Hg19. crRNAs of the six pools are shown in distinct colors.

Suppl. Figure 3

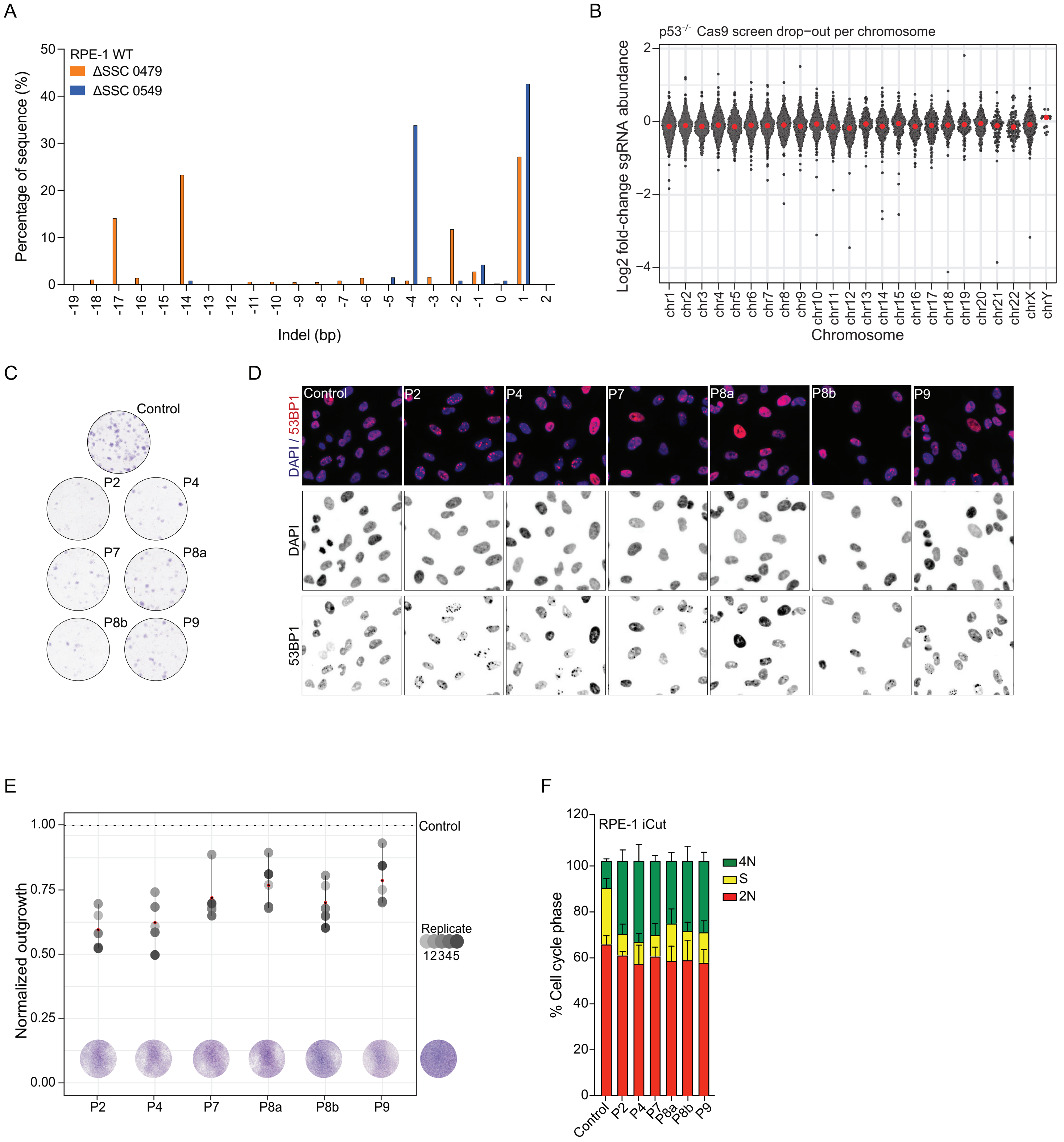

Supplement figure 3

A) Insertions and deletions of RPE-1 ΔSSC_0479 and ΔSSC_0549 cells, determined by TIDE (38). Note the complete loss of wild-type sequence. B) Fold-change of sgRNA abundance in $p53^{-/-}$ screen plotted per chromosome. The red dot indicates the mean fold-change of all sgRNAs targeting the same chromosome. C) Representative images of crystal violet staining of colony-forming assay 7 days posttransfection with the indicated crRNA pools. D) Representative images of 53BP1 staining 24h after transfection of control and pooled crRNAs, labeled with DAPI for nuclear staining. E) Normalized outgrowth of RPE-1 iCut cells 7 days after transfection with the indicated crRNA pool. Images show cells after fixation and crystal violet staining. Mean ± s.d. of at least 3 biological experiments. F) Cell cycle profile of cells 24h after transfection with control or pools of crRNAs, based on PI staining. Mean ± s.d. of at least 3 biological experiments ($n \geq 1000$ cells measured per experiment).

Suppl. Figure 4

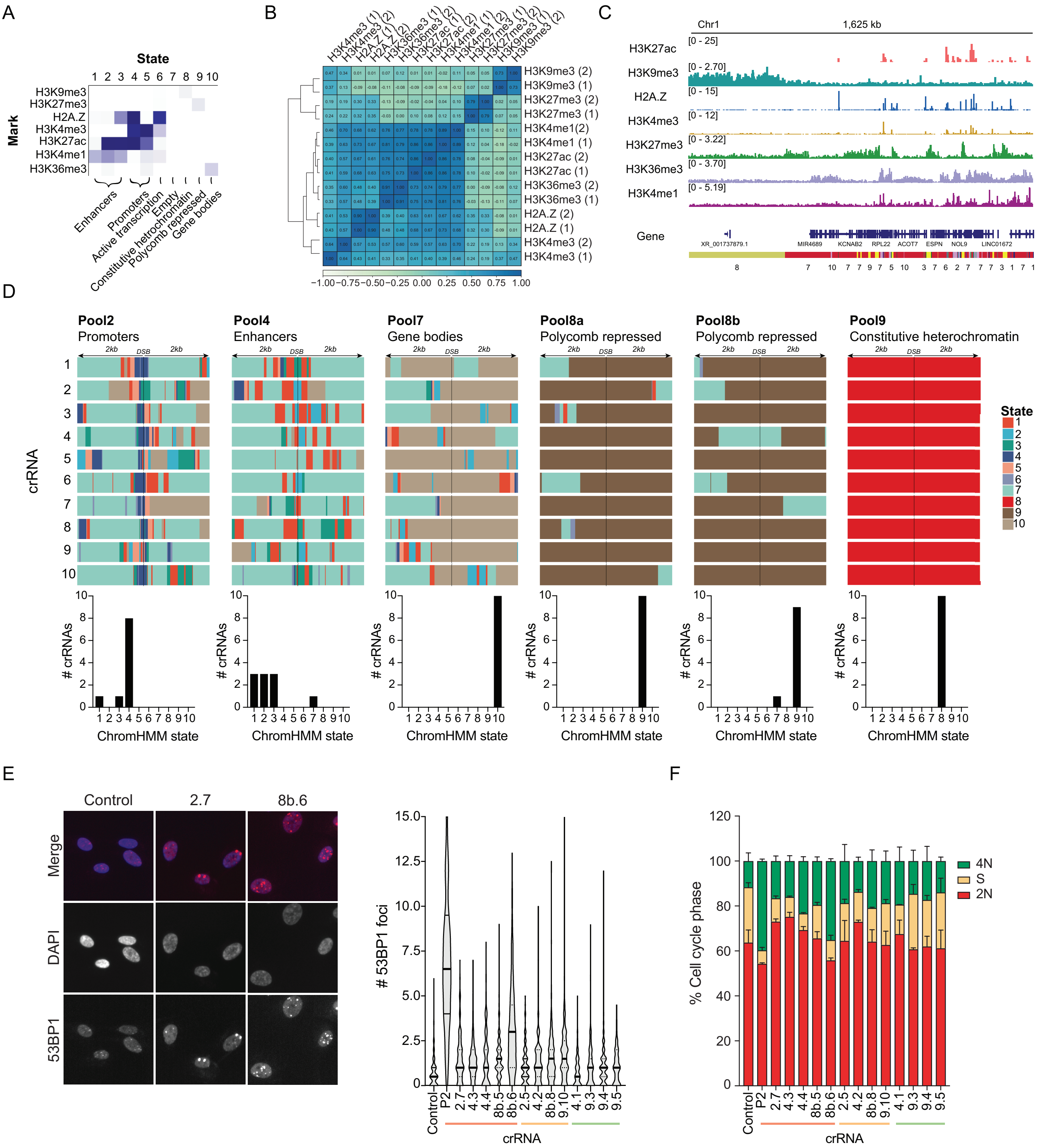

Supplement figure 4.

A) ChromHMM analysis of 10 user defined states of chromatin in RPE-1 cells including extra ChIPsequencing tracks (H2A.Z, H3K27ac, H3K9me3) aligned to Hg38. B) Correlation of the read-counts (10kb bins across the genome) of two independent replicates of ChIP-sequencing of the indicated histone-marks aligned to Hg38. C) H3K27ac, H3K9me3, H2A.Z, H3K36me3, H3K4me1, H3K4me3, and H3K27me3 ChIP-sequencing tracks from the p-ARM of chromosome 1 in RPE-1 cells aligned to Hg38. Numbers in the bottom refer to the ChromHMM states that were determined for this region. D) Reanalysis of all single crRNAs from the pools originally classified in states defined from Hg19ChromHMM. All crRNAs (except for crRNA 2.2/2.5/8b.4) target the same ChromHMM state as defined before using Hg38-ChromHMM. E) Representative images of 53BP1 staining of cells transfected with control and single cutting crRNAs, labeled with DAPI for nuclear staining, fixed 24h after transfection. Quantification of the number of 53BP1 foci for every individual crRNA (cells were analyzed from three independent experiments). F) Cell cycle profile of cells 24h after transfection with control or single cutting crRNAs, based on PI staining. Mean ± s.d. of at least 3 biological experiments (n ≥ 1000 cells measured per experiment).

Supplement table 1: SCC library

List of sgRNA sequences with corresponding name and genomic location.

Supplement table 2: Screen data 8 vs 4 days

Data of screen analysis of RPE1 WT -Cas9, WT +Cas9 and $p53^{-/-}$ at day 8 compared to day 4.

Supplement table 3: Screen data 16 vs 4 days

Data of screen analysis of RPE1 WT -Cas9, WT +Cas9 and $p53^{-/-}$ at day 16 compared to day 4.

Supplement table 4: crRNA and primer sequence

A) Sequence of crRNAs in different pools. B) Primer sequences used for this study. C) Sequence of shifted crRNAs.

Supplement table 5: sgRNA locations Hg19 and Hg38 per chromatin state

List of sgRNAs from the screen (see Suppl. table 1) with annotated location in Hg19 and Hg38 genome assembly. Including annotation of chromatin state as determined using ChromHMM output.
