# Supplementary material for: Double-strand break toxicity is chromatin context independent
Source: Nucleic Acids Res. 2022 Sep 15;50(17):9930–47. doi: 10.1093/nar/gkac758 (PMC9508844; doi:10.1093/nar/gkac758)
Supplement: gkac758_Supplemental_Files [file gkac758_supplemental_files.zip › Supplement table 4. crRNA and primer sequence.docx]

Supplementary table 4a: crRNAs pools

| Pool | Name | Sequence | Location |
| --- | --- | --- | --- |
| P2 | 2.1 (SSC_1570) | AACCAGCGCGACCTCCCAGC | Chr12: 89747160 |
| P2 | 2.2 (SSC_2021) | CCGCTGCTATAAATAGGGCG | Chr1: 6662945 |
| P2 | 2.3 (SSC_3814) | AGCCCTGATTGGATGGGACG | Chr7: 139045203 |
| P2 | 2.4 (SSC_4684) | TCTTCTTCGGAGTCCAGATC | Chr6: 119214631 |
| P2 | 2.5 (SSC_1311) | ATAGATATCCGCAGACTGGT | Chr19: 42773306 |
| P2 | 2.6 (SSC_3329) | GCCCCCGGAGGAGGCGGTGT | Chr5: 96272198 |
| P2 | 2.7 (SSC_4028) | CATTCCTCGAAAGAGGGCAA | Chr12: 104324465 |
| P2 | 2.8 (SSC_4858) | AAGGAACGCGGCCCCGGCGG | Chr7: 139044140 |
| P2 | 2.9 (SSC_5242) | AGCCCCCAGAGCCTCCCAAG | Chr17: 1620271 |
| P2 | 2.10 (SSC_5518) | TTTTGCCCGAGCCTTTCCCT | Chr17: 38256859 |
| P4 | 4.1 (SSC_2580) | TCCGCCTGCTTTACATAATA | Chr14: 73929568 |
| P4 | 4.2 (SSC_3331) | ATTGATAGAGGATTTGGAAT | Chr8: 77612269 |
| P4 | 4.3 (SSC_3394) | CAATTTGTGATGGATATCTC | Chr7: 98701485 |
| P4 | 4.4 (SSC_3480) | GATTTTAACTGTTTCCCACC | Chr10: 92949504 |
| P4 | 4.5 (SSC_3958) | TCTGGAAGTGGGATTTATAC | Chr12: 96753865 |
| P4 | 4.6 (SSC_0291) | CAGCTATCAATCAAAGATCT | Chr3: 159847285 |
| P4 | 4.7 (SSC_4528) | CACATCTATTGAACTTCAGT | Chr1: 172421381 |
| P4 | 4.8 (SSC_4595) | AAGCACCACACATTTCTGGC | Chr10: 80724278 |
| P4 | 4.9 (SSC_0113) | CACTGGAAAATTCCAGGCAT | Chr1: 90417442 |
| P4 | 4.10 (SSC_0290) | GGATTGCAGGTCATTTCTCA | Chr6: 11970387 |
| P7 | 7.1 (SSC_6171) | AATGGCAATTTAGAACTCAT | Chr15: 50874650 |
| P7 | 7.2 (SSC_3199) | CAGTCCCGATGGGTTTTCCA | Chr4: 2942958 |
| P7 | 7.3 (SSC_3344) | AAGATGATTATAGGTGTAAT | Chr14: 69341994 |
| P7 | 7.4 (SSC_3680) | AGGTCAGCCCACCCCCAGTT | Chr9: 36210893 |
| P7 | 7.5 (SSC_3718) | ACCTAAATCTCTGTCATGGC | Chr4: 187522219 |
| P7 | 7.6 (SSC_4131) | TGTATAACTAAGGGTAGGTG | Chr10: 5835647 |
| P7 | 7.7 (SSC_4247) | AACAAAGATGTTCACTACCA | Chr19: 24274250 |
| P7 | 7.8 (SSC_4612) | AAAGTCCATATGTACTTACC | Chr9: 134323231 |
| P7 | 7.9 (SSC_4718) | TGCGGCGTTATTTTTAAGCT | Chr12: 120988704 |
| P7 | 7.10 (SSC_4922) | GATGCATGGTGAGCTTTTCC | Chr5: 146777516 |
| P8a | 8a.1 (SSC_0019) | TACCCTGTTCCTGCATCAGA | Chr1: 25203267 |
| P8a | 8a.2 (SSC_0041) | TGTTTGTGTGCCTCATCAAT | Chr20: 36583705 |
| P8a | 8a.3 (SSC_0289) | GGCGGGCTGGTCTCCCCTTG | Chr6: 41410873 |
| P8a | 8a.4 (SSC_0338) | GGATGAAGCTCTCCTCTCTG | Chr9: 128990478 |
| P8a | 8a.5 (SSC_0718) | TACACTACAGTGAGAACTTG | Chr10: 72676839 |
| P8a | 8a.6 (SSC_0952) | TATTGAGCATACGGGTGCAA | Chr11: 68414668 |
| P8a | 8a.7 (SSC_2339) | CCTCGGCTCGGGGCTGCGGC | Chr7: 1269468 |
| P8a | 8a.8 (SSC_2610) | GCTATTGGCACAGTTGGCTG | Chr14: 93224862 |
| P8a | 8a.9 (SSC_3274) | CTGGAGAAAATGGGATACCA | Chr12: 53095727 |
| P8a | 8a.10 (SSC_3460) | CTGTTGAGGCCAGAATAGAA | Chr15: 51990220 |
| P8b | 8b.1 (SSC_1198) | TGCACCTCCGGGACCACGCG | Chr8: 11517979 |
| P8b | 8b.2 (SSC_1948) | TACGTGTTGTAGCCGTGTTG | Chr10: 2921769 |
| P8b | 8b.3 (SSC_2218) | TCTCTGACAAAACAGACTAA | Chr12: 8678069 |
| P8b | 8b.4 (SSC_0073) | ACCAGGTTGCCTTCAGGGCA | Chr4: 5918229 |
| P8b | 8b.5 (SSC_1682) | TTCTGGGACGAGGATTTCGG | Chr13: 27316360 |
| P8b | 8b.6 (SSC_2509) | CAGTAGGTCATGGGGGGAGT | Chr16: 9266426 |
| P8b | 8b.7 (SSC_2981) | CTGTATGAGACTGCATATAA | Chr1: 18296813 |
| P8b | 8b.8 (SSC_3622) | CTCTGTAATCAGCCTGGACG | Chr20: 17418663 |
| P8b | 8b.9 (SSC_3650) | ACATGGTCAGGAATGACCCA | Chr5: 6482899 |
| P8b | 8b.10 (SSC_3776) | ACTTAATATTGTGCTCTATA | Chr3: 11080545 |
| P9 | 9.1 (SSC_0582) | GGTCGTCACCTGATGAGTGT | Chr19: 22610430 |
| P9 | 9.2 (SSC_0959) | GAGTTTTTGCCAGCTCGCAC | Chr8: 47005327 |
| P9 | 9.3 (SSC_1042) | GCGTCTCTCGTTTTCTTTGC | Chr10: 133357195 |
| P9 | 9.4 (SSC_2059) | GTTTTAAGCCGCGGCGTTGG | Chr22: 49680562 |
| P9 | 9.5 (SSC_1357) | CAACATCTGGGTACTGCCTG | Chr2: 129946586 |
| P9 | 9.6 (SSC_1604) | TCCATTTAATGTTATCCCCT | Chr18: 76266575 |
| P9 | 9.7 (SSC_2100) | ATTCCTGTCTGAGTCTGGGT | Chr18: 73572870 |
| P9 | 9.8 (SSC_2373) | GGAGGTGCAGGACCCCACGT | Chr17: 21943753 |
| P9 | 9.9 (SSC_4062) | TAGGGAATGAGAACCGCGAG | Chr8: 983674 |
| P9 | 9.10 (SSC_5455) | CTGCTCCTCAAAGGAGGCGG | Chr15: 28042267 |

Supplementary table 4b: Primer sequences

| crRNA location | Sequence primer fwd | Sequence primer rev |
| --- | --- | --- |
| 2.5 (SSC_1311) | CTTTCATTTCATTGGCTACTG | CTGTCACCCTGATCTAGTCC |
| 2.7 (SSC_4028) | GGGAAGTGGGGGTGAAAAGC | TATGCAACCTCTGGCGAGGG |
| 4.1 (SSC_2580) | TGCTTTCACCACCGGGAAG | ACACAGTTGGTAAATGGCAG |
| 4.2 (SSC_3331) | AAGCCTCAATGTGGCTTTAGC | CCTCTTCTACGGTCCAGTGA |
| 4.3 (SSC_3394) | CCAGAGTCTTTTCATAACCAG | GGTTCATGCCCATAATCGC |
| 4.4 (SSC_3480) | TCTACACTTGCTGTTTGAGG | GCTTTGTTGTAGCAGCAC |
| 8b.5 (SSC_1682) | AAAAGTGTGCGATGGGGTAG | CAAGCATAGGGTGGAAGCAT |
| 8b.6 (SSC_2509) | TTCAATGCACACACTTGAAGG | CCACGCCTGACTGATTTTTG |
| 8b.8 (SSC_3622) | CAACTCCTTTACTACGCTTGG | GCCTGCGTTAAACATAGCGTTC |
| 9.3 (SSC_1042) | CAATGTTTCACTGCACAAGG | TCTTGAGGTAGGTGTCCAAG |
| 9.4 (SSC_2059) | CGATGCTCAGTGTCATCCGC | ATGGAGAGGGCAAAATAGCC |
| 9.5 (SSC_1357) | CATCTAAGCCAAGTGTCGGG | TACTGAGCACCATCATCCTCC |
| 9.10 (SSC_5455) | AGCTCTCTGGCAACCCTCTG | TAGACACTTAAGCTGCAATGC |

Supplementary table 4c: crRNAs shifted

| Name | Sequence |
| --- | --- |
| 2.5S | ACCAATAGATATCCGCAGAC |
| 2.7S | CGTCACCATTCCTCGAAAGA |
| 4.1S | CAAGCCATATTATGTAAAGC |
| 4.2S | TGACATATTGATAGAGGATT |
| 4.3S | ATATGAAAAGCAATTTGTGA |
| 4.4S | CTTAGGCCTTCTTGCCTGGT |
| 8b.5S | ATTTTCTGGGACGAGGATTT |
| 8b.6S | TTTAAACAGTAGGTCATGGG |
| 8b.8S | AGGACTCTCTGTAATCAGCC |
| 9.3S | CGTCTCTCGTTTTCTTTGCT |
| 9.4S | ATTGTTTTAAGCCGCGGCGT |
| 9.5S | GGATTTCAAGAGGCAACATC |
| 9.10S | CAGCTGCTCCTCAAAGGAGG |
